# Graph‐Theory Approach to Element Miscibility and Alloy Design

**DOI:** 10.1002/advs.202521018

**Published:** 2025-12-20

**Authors:** Andrew Martin, Kien Nguyen, Sebastian Zaatini, Michael Lastovich, Bharat Gwalani, Paul Bogdan, Martin M. Thuo

**Affiliations:** ^1^ Department of Materials Science & Engineering North Carolina State University Raleigh North Carolina USA; ^2^ Ming Hsieh Department of Electrical and Computer Engineering University of Southern California Los Angeles California USA

**Keywords:** alloy design, elemental interaction, interface design, miscibility, network theory

## Abstract

With 118 currently known elements, hence millions of possible quinary combinations, understanding and discovering potentially beneficial mixtures is challenging. Possible stable mixtures, however, are vital for alloy design or realization of new materials. Thermodynamically favorable interactions can also lead to enhanced materials properties. We apply graph theory to map relation in key thermodynamic parameters between elements, therefore revealing possible favorable (miscible) pairs and their congeners. Using closeness centrality and Lipschitz‐Hölder exponent, we define miscibility across the whole period table, albeit at ambient conditions. Whereas most metals have fair solubility, hyper‐ and hypo‐ centrality (high and low solubility respectively) clusters are identified. We confirm our graph‐based approach in understanding miscibility by comparing results to CALPHAD and Miedema's model. Unlike the heuristic models, the graph‐based approach is amenable to machine learning enabling prediction at extreme environments by extrapolating known scaling laws.

## Graphical abstract

Graph and network theory enables pathway toward complex multiscale interactions between different elements for alloy design or interface engineering. Utilizing element's inherent properties and preferential interactivity, favorable mixed material formation, solubility and miscibility can be predicted. Network clusters ease the identification of elements with higher/lower degree of interaction from large groups of elements.

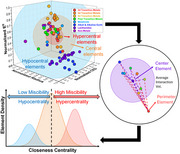

## 1 Introduction

Interaction and miscibility between elements are critical pieces in the pursuit of novel material discovery [1, 2]. Theories and models exploring preferential reactions [3, 4, 5], miscibility rules [6, 7, 8, 9], energy landscape approximation [10, 11] and intermetallic formation [12, 13, 14] have provided great insights on how different elements interact. Empirical theoretical analysis of these models have resulted in modern predictive materials design tools, however, fully quantitative miscibility and interaction parameters have yet to be realized [15, 16, 17]. Complexity in real material systems (beyond static equilibrium phase space) [18] further limits the degree of predictability in materials and process designs, requiring higher dimensionality to map out non‐equilibrium variables [19, 20]. Dependence of a material property on its structure necessitates fundamentally understanding each constituent element's thermodynamic properties, chemical characteristics, and their interactions for accurate mixing/reaction prediction. Given the large number of known elements, understanding each element's property(ies) and possible multi‐element interactions is challenging due to the sheer number of combinations (e.g., for quinary alloys, *C*(118,  5) ≈ 1.75 × 10^8^). Advances in network and graph theory, however, has resulted in better, amicable tools to understand and analyze complex systems [21, 22, 23, 24, 25]. The ability to map large scale interactions, simplify the information density landscape and highlight potential correlations across unknown pairs or clusters through calculable graph parameters [26, 27, 28] provide a path to understanding elemental interaction, hence, structure and interface formation across the periodic table [29, 30, 31, 32]. Similar approach of isolating binary descriptors has recently been utilized for catalytic selectivity, which brought upon novel methodology for catalyst design and predictability [33]. This work explores the application of graph theory to generate inter‐element interaction network by considering felicitously chosen element parameters (based on past and recent theories) [3, 8] to extend the Preferential Interactivity Parameter (PIP) beyond the limits of 3 dimensions [34].

**FIGURE 1:**
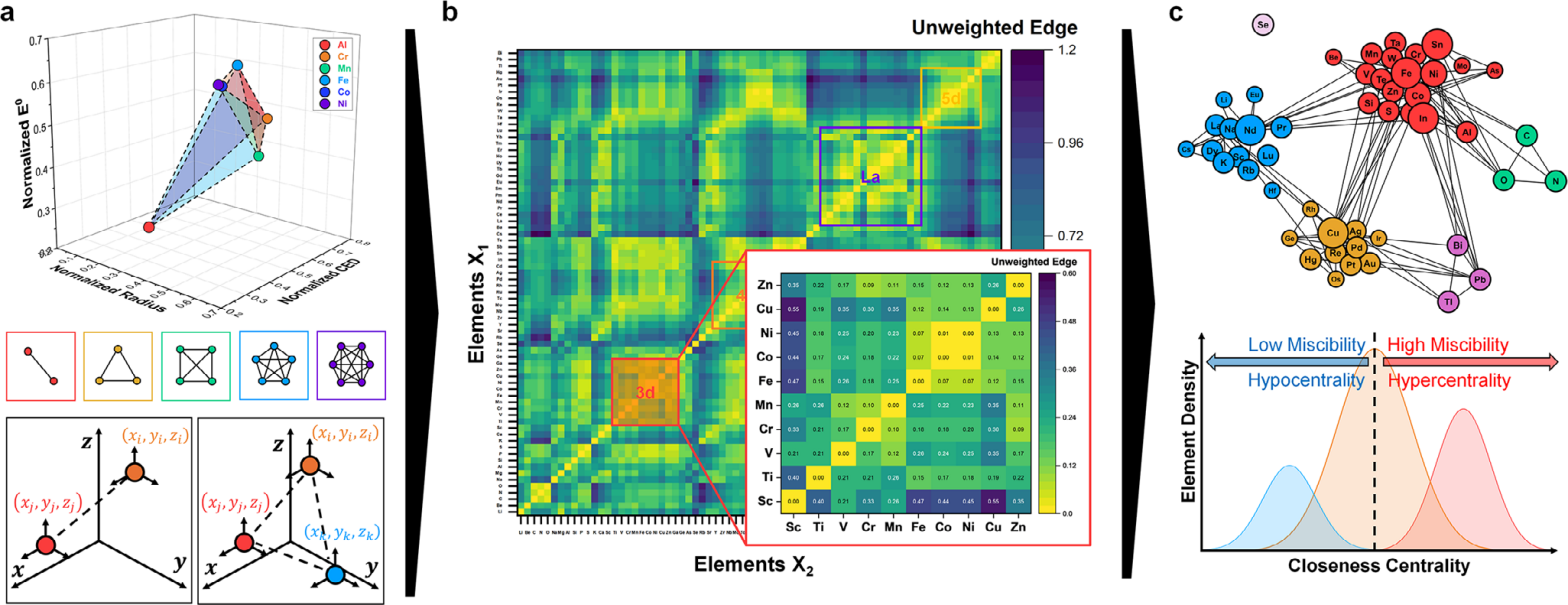
Graph analysis of coupled elemental interaction. (a) Graph representation in Preferential Interactivity Parameter (PIP) and schematic of possible graph architectures between available nodes (elements). (b) Unweighted edges heatmap capturing binary interactions from a 72 elements PIP. (c) Schematic of generated network and its analysis based on closeness centrality as the key metric revealing clustering into high, medium, or low miscibility.

Preferential Interactivity Parameter (PIP) [34] emerged as the perfect platform on which to adopt graph theory to predict miscibility in part due to its geometrical foundation (Figure S1) [3]. Building upon the Hume‐Rothery rules and following theories [8], PIP adopts all quantitative thermodynamic variables and expands the 2D view of miscibility (based on size and electronegativity) to four dimensions (3D cartesian coordinates plus partitioning between bulk and surface) by considering cohesive energy density (CED), diffusivity (atomic size), reactivity to the external environment (redox potential), and bulk‐to‐surface partitioning criterion [3, 30, 34]. Utilization of graph theory to represent interactions between 2 or more elements as edges (Figure 1a) results in a heatmap where regions of preferential interactivity can be evaluated, allowing for easier (mathematics‐assisted) navigation of the interaction landscape (Figure 1b; Figure S2). Analysis of these inter‐elemental networks reveals groups and clusters of potential elemental pairing, as well as global or local miscibility of elements (Figure 1c). Previously, this approach has been utilized to predict surface speciation and order in complex multi‐elemental systems such as high entropy alloys (HEAs) [30], metastable materials [34], liquid metals and separation [3, 8]. Quantification through graph and network metrics, however, is yet to be performed. Application of networks increases accessibility in complex materials design with important implications in mixing, separation, interface design, passivation, structural property enhancements and discovery of novel materials [32, 35].

## 2 Results and Discussion

**FIGURE 2:**
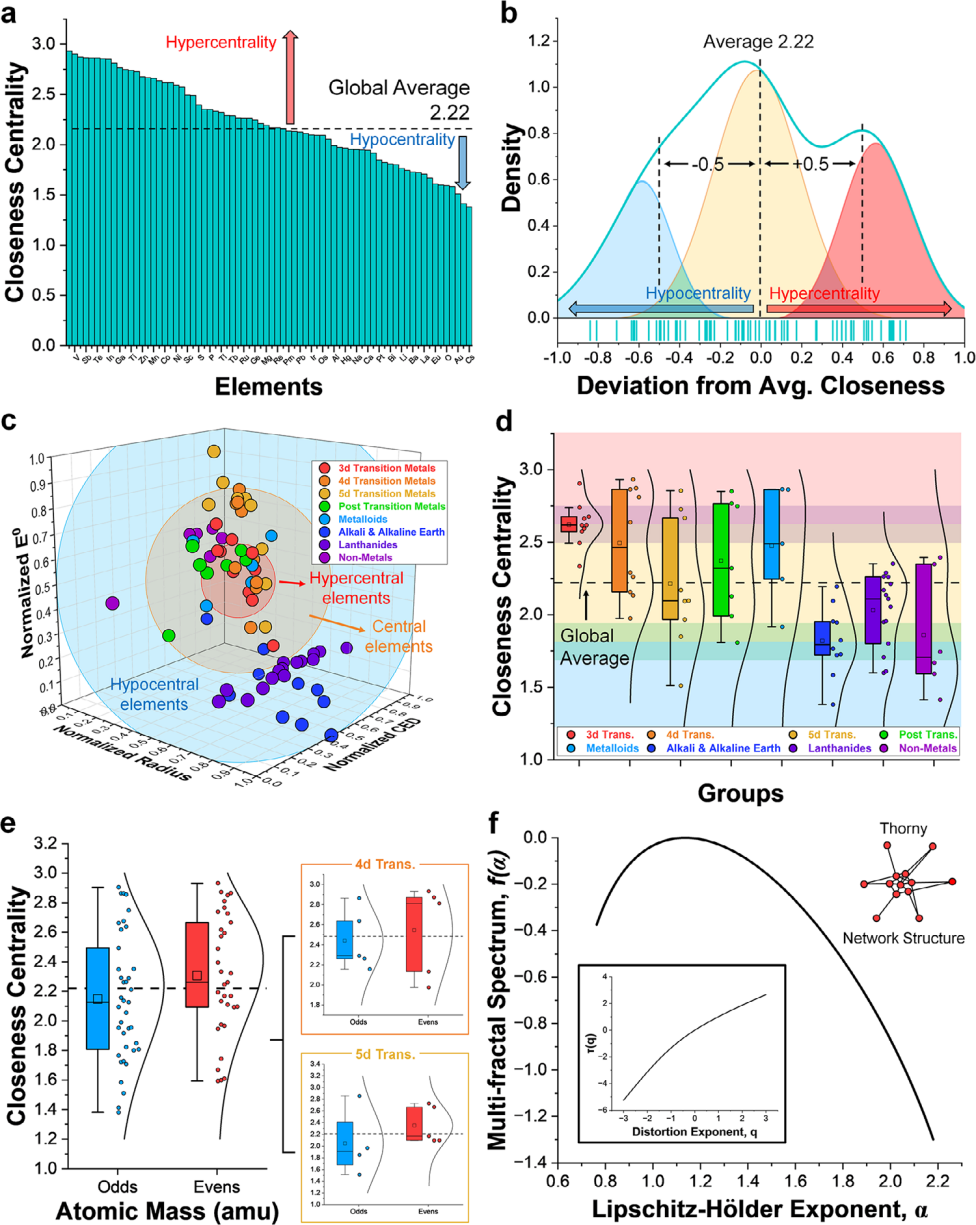
Graph analysis of Preferential Interactivity Parameter. (a) Global closeness centrality values of 72 elements analyzed in this work. (b) Trimodal distribution of closeness centrality standard deviation indicating distinct groupings of hypo‐central, central and hyper‐central elements. (c) Schematic representation of how the different groups manifest in PIP's 3d space. (d) Statistical analysis of different elemental group's closeness centrality values. (e) Statistical analysis of closeness centrality based on odd or even amu atomic masses. (f) Calculated multi‐fractal spectrum and Lipschitz‐Hölder exponent based on node‐based multifractal analysis (NMFA) indicating a thorny network structure (insert: measured distribution exponent and its mass exponent).

Node‐based graph analysis is performed on 72 element Preferential Interactivity Parameter (PIP) using the 3‐D Cartesian coordinates for each element. Several groups such as halogens, noble gases, actinides, and radioactive elements are not included in this analysis due to incompatibility or limited experimental data. The Cartesian coordinates (x, y, z) correspond to each element's relative (to other known elements) covalent radius, cohesive energy density (CED), and standard reduction potential (E^0^) respectively. These three elemental characteristics are chosen as previously described [34], where covalent radius captures the effect of element's size toward diffusivity based on Stokes‐Einstein‐Sutherland equation (*D*∝1/*r*), under the assumption that the element is not charged [36]. The CED captures the element's pull toward itself, depicting resistance to escape an initial state. Standard reduction potential, E°, captures an element's propensity to oxidize or reduce upon interaction with other elements or ambient environment. The E^0^ is utilized inlieu of electronegativity (Hume‐Rothery rules) as it is well defined and utilized in literature and enables prediction of partition under redox conditions. As a graph, these coordinates are considered nodes and their distances to other neighboring elements are defined as edges (equal contributions, equimolar). For each axis within the PIP diagram, a finite‐sized (size depends on n‐elements) adjacency matrix can be created where the edges for each axis (*E_x,y,z_*) are represented in the matrix as:

(1) $$E_{x,y,z}={\left[\begin{array}{ccc} d_{ii} & \text{…} & d_{in}\\ \vdots & \ddots & \vdots \\ d_{ni} & \text{…} & d_{nn} \end{array}\right]}_{x,y,z}$$

where *d_ij_* is the distance between the coordinate of element *i* and *j*. From these edge values, the total 3‐D unweighted edge is calculated using Pythagorean theorem. This global adjacency matrix (Table S1) is used to calculate network metrics such as closeness centrality, degree centrality, clustering coefficient and fractal dimension (see Methods) from the PIP system. The global closeness centrality calculations of all 72 elements display a relatively normal distribution with an average value of 2.22 (Figure 2a; Table S2). Evaluating this normal distribution through each element's deviation from the average, however, reveals a trimodal distribution (Figure 2b) that shows distinct grouping of highly centralized nodes/elements (hypercentrality), intermediates, and those that are not centralized (hypocentrality). Whilst majority of the elements are within the intermediate group, the hypercentral and hypocentral elements are of a particular interest due to their potential implications in bonding and mixing tendencies. Physically, the hypercentral elements sit close to the other nodes (shortest distance to nearest neighbors), sharing similar properties (atomic size, electronic and structural) and hence, would likely be miscible in a mixed system with most elements. Thermodynamically, this is in support of the minimization of chemical and structural incompatibility (energetic differences and lattice mismatches/strains) [32], hence their configurational entropy and overall free energy. Hypocentral elements are the exact opposite where their properties differ significantly with others, requiring specific elemental groups or intermediates to support its miscibility. This is not a negative feature, however, as some elements are useful in interfacial engineering and design for passivating layers. Both aspects have their benefits in different applications and can see different interpretations from different users, whereas hypocentral elements may be considered thermodynamically to be stable interface formers. These different element groups have a clear visual representation in the 3‐D PIP space (Figure 2c), which provides simplicity on how different elemental groups are distinguished.

Analyzing the different elemental clusters reveal the dominant contributors to the centrality groupings, where most transition metals (from 3d to 5d including post transition) and metalloids have higher than average centrality values while the other groups (including rare earths) have lower than average centrality (Figure 2d). This distinct split between transition metals and other elements relates back to differences in electronic (redox) properties and orbital configuration. The electron configuration of transition elements (partially‐filled valence shell) leads to their ability to interact with other elements more readily than those in the early/late periodic groups [37]. This finding bears parallels to the different bond properties as captured by the van Arkel–Ketelaar triangle [38, 39], where the boundary in different bond‐type can be interpreted using centrality terms (metallic bond refers to low electronegativity difference akin to hypercentral elements). Further investigation on the distribution based on odd and even atomic masses reveal and odd‐even parity effect, where odd atomic mass elements possess lower than average closeness centrality values whilst evens cogners show higher closeness centrality (Figure 2e). This mass parity effect is also shared in smaller elemental groupings (e.g. 4d and 5d transition metals‐ selected due to their broad distribution, Figure 2d) where the contributors for hypercentrality effects are dominated by the even mass elements and vice versa. We infer that this odd‐even parity effect is caused by the nature of the nuclei, which, due to its binding properties leads to a pairing gap between those with odd and even mass numbers [40]. Graph theory, therefore, allows us to reveal the role of the nucleus in element interactions and therefore miscibility, pointing to potential influence of the specific gravity of the nuclear (atomic relativistic effects) [41]. Evaluation of other network metrics such as degree‐centrality and clustering coefficient (Figures S3 and S4 and Tables S3–S5) reveals similar behavior, albeit with a higher bimodality between the hyper‐ and hypo‐central components. Node‐based multifractal analysis (NMFA) was also performed on the inter‐elemental network (Figure 2f), where the fractal dimensions can be analyzed. The multi‐fractal characteristic is apparent from the non‐linear trend in mass (*τ(q)*) vs distortion exponent (*q*) (Figure 2f inset) and analysis on the multi‐fractal spectrum (*f(α)*) further confirms the thorny structure of this inter‐elemental network. Given that hypocentral elements exist in the perimeter of the overall network cluster, this multi‐fractal dimension further supplements the interconnectivity of these elemental interactions.

**FIGURE 3:**
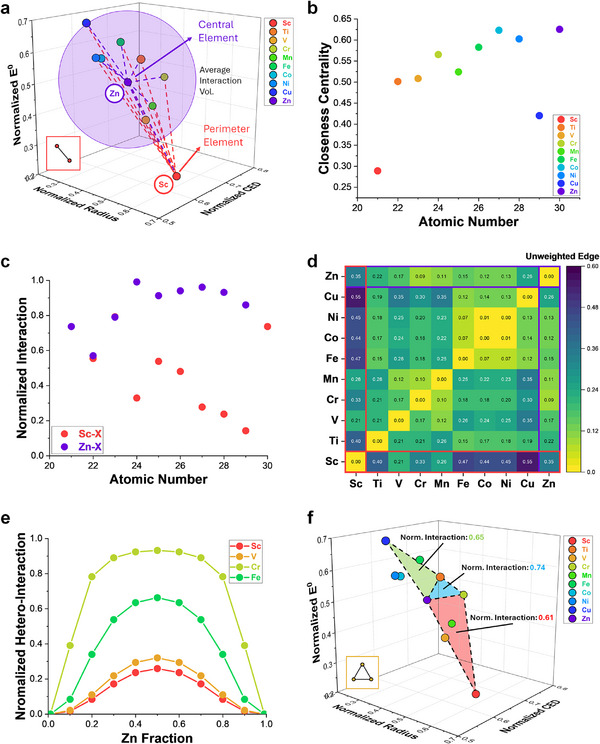
Graph analysis of interactions between 3d transition metals. (a) Preferential Interactivity Parameter of 3d metals (lines indicate edges between elements (red for Sc‐X and purple for Zn‐X). (b) Calculated closeness centrality values of each 3d metals binary combination and (c) calculated normalized interaction of Sc and Zn with other metals in the 3d group. (d) Unweighted edge heatmap matrix of 3d transition metals. (e) Depiction of ternary interaction between 3d metals (Green: Cu‐Ti‐Zn, blue: Cr‐Ti‐Zn, red: Sc‐Cr‐Zn). (f) Calculated quaternary interactions of equimolar Fe‐Cr‐Mn‐X alloys and their graph representations.

To demonstrate the implementation of graph analysis on a smaller scale (local groups), PIP and the adjacency matrix were divided into specific key groups (Figure S5). This methodology, however, can be applied to any combination of elements (not limited to specific periods or groups). For brevity, however, a sequential homologous group (3d transition metals) was used to demonstrate this analysis (Figure 3a). In a smaller group, distinction between an element with relatively short edges (high centrality, Zn) vs one with long edges (low centrality, Sc) can be easily seen. Here, it can be considered that Zn has a relatively high solubility with its 3d neighbors due to its proximity and similarity in properties. From this distinction Zn can be considered as a central element, whilst Sc is a perimeter element, this is reflected in the discrepancy in the local closeness centrality value of Zn versus Sc (Figure 3b). Note that these edge values should not be taken based on their immediate face values (i.e. close proximity/small number = good interaction and vice versa), rather, its utilization and interpretation depend on the use‐defined weight of these inter‐element connections. In this work, the topic mainly focuses on miscibility, hence, the emphasis on atomic similarities to minimize lattice strains and maximize chemical compatibility. For other purposes such as interfacial engineering where the opposite may be of interest, however, this interpretation will change. It is also to be noted that whilst Sc may be sitting on the perimeter in this 3d group (and has a low local centrality), this may not be true when Sc is paired with other elements in different groups as seen from the global average (Figure 2d). Further, another metric such as interaction volume (*V_int_*, akin to Darken‐Gurry's ellipse) can be calculated based on the average edge distance from an element of choice to all its neighbor, hence measuring certain element's sphere of influence within a group. Here, the boundary of *V_int_* is defined by the choice of grouped elements using a fully connected network (all nodes connected), whereas in this demonstration the selected neighbors are the 3d transition metal elements to capture local miscibility within this group (Figure 2a). These volumes can then be converted into normalized interaction (*I_n_*) values (Normalized to interaction with itself, where X‐X will have a *I_n_* value of 1) where each element pair's relationship can be evaluated to determine preferentiality and favorable interactions (Figure 3c). These values are calculated based on the measured edge distances as follows:

(2) $${\bar{E}}_{x,y,z}={\left(\frac{E_{i}+\cdots +E_{n}}{n}\right)}_{x,y,z}$$

(3) $$V_{int}=\frac{4}{3}\;\pi \left(\frac{{\bar{E}}_{x}\cdot {\bar{E}}_{y}{\bar{\cdot E}}_{z}}{6}\right)\chi$$

(4) $$I_{n}=1/\left(V_{int}+1\right)\;$$

where the average edge for each axis (${\bar{E}}_{x,y,z}$) is used to calculate *V_int_* and χ is a constant scaling factor. From Figure 3c, Zn has overall higher interaction values compared to Sc, which was previously qualitatively shown in the PIP space. This diagram can alternatively be represented as a heatmap, hence distinctly highlighting favorable/unfavorable interactions between element pairs based on their unweighted edges (Figure 3d). To scale the capability of this analysis, a weighting factor is needed to take the system beyond equimolar combination. Hence, the adjacency matrix can be transformed based on the reciprocal concentration of each element (*c_i_*) within a compound,

(5) $$E_{x,y,z}\left(comp\right)={\left[\begin{array}{ccc} d_{ii} & \text{…} & d_{in}\\ \vdots & \ddots & \vdots \\ d_{ni} & \text{…} & d_{nn} \end{array}\right]}_{x,y,z}{\left[\begin{array}{c} 1/c_{i}\\ \vdots \\ 1/c_{n} \end{array}\right]}_{x,y,z}$$

With the edges weighed, the sphere of influence of individual elements can be accounted for based on how much concentration of certain elements are within a compound mixture. Coming back to 3d transition metals, the strongest and weakest connection with Zn can be clearly seen in Figure 3a. When Zn is used as a knob and concentration of Zn is modified with each binary combination, a relationship akin to a miscibility gap can be seen (Figure 3e) albeit factors such as temperature and pressure are not yet accounted for. Here, the captured values quantify hetero‐atomic interactions (coupling strengths), rather than isolated stability. These relationships display the clear distinction between the strongly interacting elements and the weak, displaying various peak heights (which may have a relationship to the free energy) and different drop off rates as concentration goes toward 0 or 1 (pure unary compound). Furthermore, given the finite scaling of the adjacency matrix, n‐number interactions (ternary, quaternary…) can be calculated simultaneously, increasing the graph dimensionality beyond binary lines. Each graphical representation provides immediate qualitative evaluation where the interaction volume can be seen based on the graph that is formed between connected nodes (Figure 3f). To further verify the validity of this graph methodology, comparison with other theoretical models for interaction preferentiality and miscibility was performed.

### 2.1 Verification with Theoretical Models and Thermodynamic Database

**FIGURE 4:**
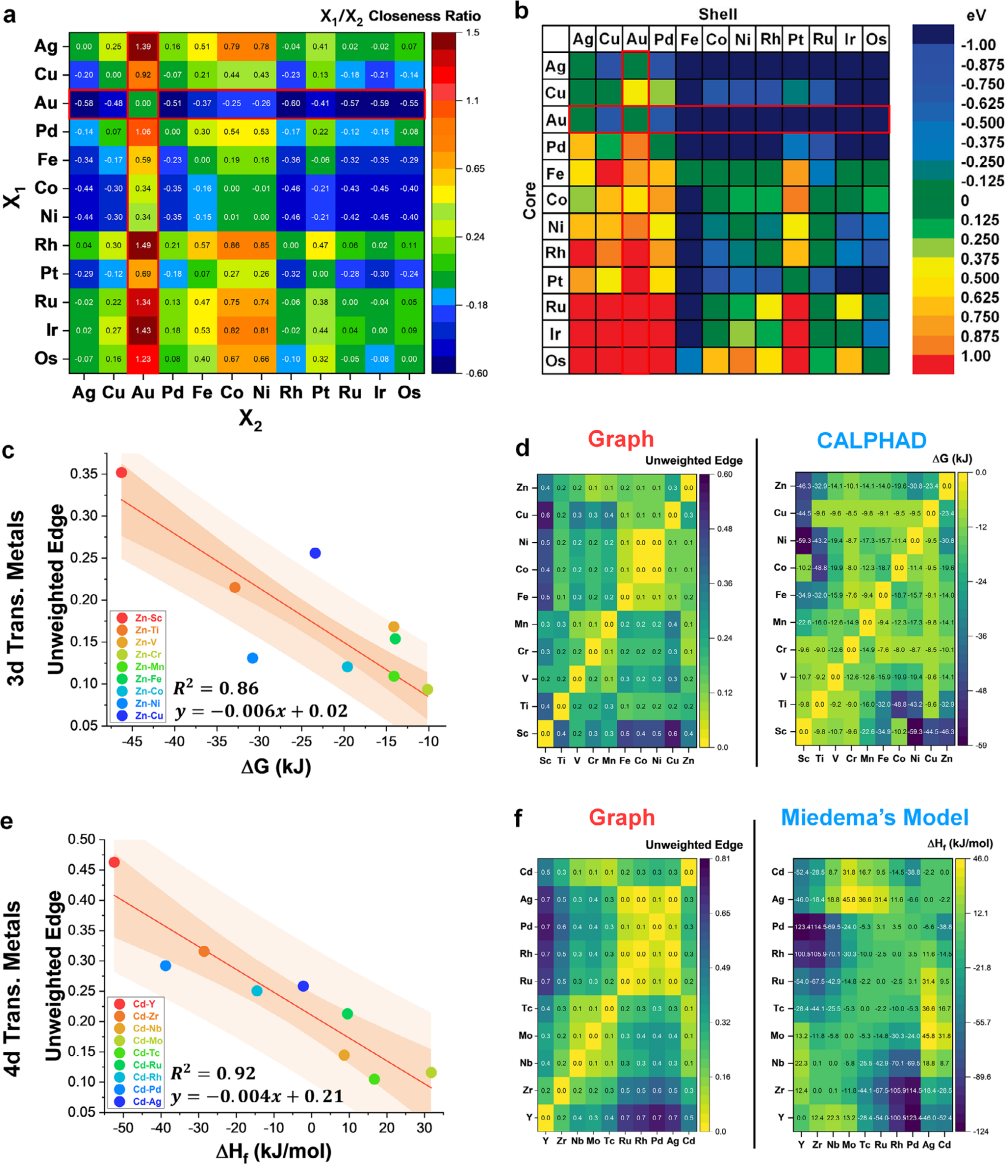
Verification and validation of graph‐based interaction measurements. (a) Calculated closeness centrality ratio of group 8 to 11 elements. (b) DFT‐calculated segregation energies of group 8 to 11 elements, ordered in terms of increasing cohesive energy. Reprinted or adapted with permission from Ref. [42]. Copyright 2009 American Chemical Society. (c) Calculated unweighted edges against total free energy calculated through CALPHAD of Zn paired with other 3d transitional metals. (d) Comparison of unweighted edge heatmap matrix and calculated free energy heatmap matrix of 3d transition metals. (e) Calculated unweighted edges against enthalpy of formation calculated with Miedema's model of Cd paired with other 4d transitional metals. (f) Comparison of unweighted edge heatmap matrix and calculated enthalpy of formation heatmap matrix of 4d transition metals.

Previously, PIP was utilized to qualitatively predict core–shell preferentiality in mixed‐element system [3, 34]. Past work [42, 43] have also suggested that this preferentiality is dependent on the interplay between each element's cohesive energy density (related to vapor pressure) and size (Wigner‐Seitz radius), both of which are accounted for within PIP. Given that miscibility and surface preferentiality in PIP is dependent on the relative centrality of each element, a ratio of the closeness centrality value (*C_i_*) for each element can be calculated to determine the core‐preferentiality (higher closeness centrality value equates to higher core preference). This ratio was taken using the following relationship (*C_ratio_* = *C*_*X*1_/*C*_*X*2_  − 1), the value was subtracted by 1 for ease of data navigation where negative values would indicate surface preferentiality and vice versa (Figure 4a). Values for groups 8 to 11 was calculated and then compared to the segregation energies (SE) measured using density functional theory (DFT) simulation performed by Wang et al. [42] (Figure 4b) where it displays similar trends in most of the 132 binary combination. In particular, Au is highlighted here as the element that has the highest surface preferentiality and the *C_ratio_* accurately resembles the trend in SE with each pair, albeit at a different number scaling.

Furthermore, since PIP still follows the classical miscibility theories by expanding upon Hume‐Rothery rules [6, 12] and their derivatives [7, 14, 44, 45], the output of this graph approach can be further verified with a widely used thermodynamic database from Thermo‐Calc using the Calculation of Phase Diagrams (CALPHAD) method [46]. The 3d transition metal group is again taken as an example for this verification. The measured edges are compared to the approximated free energy of binary equimolar combinations calculated through CALPHAD, where it displays a relatively linear relationship (within 90% confidence interval) between Zn and other 3d elements (Figure 4c). The edges, however, seem to be inversely proportional to the free energy, where high free energy is related to a low edge value. Here, we speculate that the relationship between graph and the thermodynamic model/phase diagram is inverted, where $\Delta G\approx 1/\bar{E}$. Interestingly, observing the edge values and approximate free energy of the entire 3d group displays a high degree of qualitative similarities based on their intensities and regions where high/low edge and free energies are observed (Figure 4d).

Additionally, other theoretical model such as Miedema's model [10, 11] have also been used to predict mixing properties and alloying feasibility based on the calculated enthalpy of formation (*ΔH_f_*) between combination of elements. To further supplement the verification from CALPHAD's free energy approximation, the *ΔH_f_* values were calculated using the Miedema calculator [47]. Here, the unweighted edge values were compared to the calculated *ΔH_f_* values. Cadmium was taken from the 4d transition metal group to capture trends within the group (Figure 4e). Cadmium's unweighted edges display similar linear relation with *ΔH_f_* when paired to other elements in the period, akin to the free energy approximation. The trend of the entire 4d group also displays similar behavior to CALPHAD data (Figure 4f). This further confirms the inverse proportionality between graph metric and thermodynamic values. Both theoretical models, however, do not consider the role of surface (non‐expansive) work and other external perturbations that may affect the total free energy of a system. Further investigation is therefore needed to verify the accuracy of this approach. The need for higher dimensionality in modeling and design is, however, becoming more apparent as we move from ideal conditions into real and dynamic systems with the goal of enhancing our prediction capabilities. The current approach is still limited to static elemental attributes in its ability to predict pair and coordination‐based behavior. As we move toward higher dimensional space, however, additional factors (accounting for larger, structural factors such as bond characteristics, crystal structure, relativistic effects, and others) may be introduced into this model to improve prediction in real interacting systems and under wide range of environments.

## 3 Conclusions

This work demonstrates the power of networks/graphs and graph analysis in expanding a thermodynamics‐based miscibility tool, PIP, to extend beyond limitations of 3D visualization. Relying on graph parameters, potential interactions can be revealed across the periodic table despite clustering of different groups like the 3d, 4d, 5d or f‐block elements. The utilization of PIP considers factors beyond equilibrium thermodynamic models such as diffusivity, external environment influence, and structural energy as captured by radius, E^0^ and CED respectively. Predictions from graph theory‐based methodology agree with previously known theories and simulations but this approach has the potential to scale with changes of conditions by adopting graph scaling laws. This work also reveals the unprecidented role of mass number (nucleus) in elemental interactions pointing to potential effect of atomic relativistic effects previously unconsidered in mixing and demix theories. By adopting graph theory and network science, we open the door for efficient machine learning in alloy design and predicting miscibility of elements across disparate conditions.

## 4 Experimental Section

### 4.1 Preferential Interactivity Parameter

All standard reduction potential [48], covalent radius [49] and cohesive energy density (vapor pressure) [50] values of 72 elements investigated in this work were taken from known sources and handbook. These values were then normalized to represent a min‐max range from 0 to 1 for the 72 elements (Table S6 for tabulated values). These normalized values were then plotted as a 3D scatter plot, and each coordinate is recorded. These coordinates were utilized to calculate the edge values and adjacency matrix as described in Equations (1)–(5).

### 4.2 Graph Analysis

To leverage graph theory analysis on element interactivities, we construct weighted, fully connected interaction graph *G*  = (*V*, *E*) , where each node v∈V is a chemical element, and each weighted edge $e_{\mathrm{ij}}\in E$ represents the interaction strength between two elements i and j. We compute the weight $w_{ij}=\frac{1}{d_{ij}}\;$, where the distance between two elements d_ij_ is calculated from 3D coordinates of PIP system using Pythagorean theorem. The weights are then normalized by the maximum weight in the graph to preserve the scale $w_{ij}=\frac{w_{ij}}{max(w)}$. Higher weight represents stronger similarity and compatibility between elements, and vice versa. After that, several graph theory metrics are employed to analyze the network.

(Weighted) Degree centrality is sum of edge weights between a node and its neighbors, indicating strength of its local connectivity:

$$deg\;\left(i\right)=\sum_{j\in N_{i}}w_{ij}$$

Closeness centrality quantifies how close a node is to all other nodes in the network, calculated as reciprocal of the sum of the shortest path distances from that node to all other nodes:

$$Closeness\;\left(i\right)=\frac{N-1}{\sum_{j\neq i}^{j\in V}spd\left(i,j\right)}$$

where spd(i, j) is the shortest path distance between node i and node j, N is the total number of nodes. A high closeness node is well‐positioned to influence the network efficiently. In our context, it indicates strong compatibility with a wide range of elements, which could be highly potential for alloy mixture.

(Weighted) Clustering coefficient measures the degree to which the neighbors of a node are also connected to each other:

$$Cluster\;\left(i\right)=\frac{1}{deg\left(i\right)\left(deg\left(i\right)-1\right)}\;\sum_{j,k}{\left(w_{ij}w_{ij}w_{jk}\right)}^{1/3}$$

where deg(i) is the degree of node i and j, k are any neighbor pair of i. A node with high cluster coefficient indicates that it neighbors are also tightly knit, which could be favorable for forming stable alloy.

### 4.3 Node‐Based Multifractal Analysis (NMFA)

NMFA quantifies the structural complexity and heterogeneity in the network from fractal perspective. From spectrum of distortion exponent *q*, the Lipschitz–Hölder exponent α measures the singularity of network structure:

$$\alpha =\frac{d\tau \left(q\right)}{dq}\;$$

where τ(*q*) is the mass exponent between multifractal partition function and scale [28]. A higher α indicates the greater regularity. The multifractal spectrum *f*(α), obtained by Legendre transform, demonstrating the distribution of singularities:

$$f\left(\alpha \right)=q\alpha -\tau \left(q\right)$$

The shape of spectrum reflects the structural property of network. Spectrum with long tail on the left indicates the clumpy structure, while one with long tail on the right suggests the thornlike structure. The width of the spectrum (α_*max*_ − α_*min*_) measures the degree of structural heterogeneity, where a wider spectrum signifies a more heterogeneous network.

### 4.4 CALPHAD Free Energy Estimation

Additional values for system enthalpy, system entropy, and total Gibbs free energy were calculated using Thermo‐Calc software [46]. Calculations assumed an equiatomic binary mixture at a reference temperature of 300K and utilized thermodynamic data from the TCFE14: TCS Steel and Fe‐alloys database for 9 of the 10 3d transition metals. Sc is absent from the steels database, and therefore values for Sc containing binaries utilized the TCAL10: TCS Aluminum‐based Alloys Database.

## Author Contributions

The manuscript was written through contributions of all authors. A.M. and M.T. conceived and designed the experiments. A.M. performed the experiments, analyzed and visualized the data. S.Z. performed graph experiments, K.N. performed network analysis and M.L. performed CALPHAD analysis. M.T. supervised the project. A.M., P.B., B.G., and M.T. wrote the draft and final version of the manuscript.

## Funding

This work is supported by North Carolina State University through startup funds and by the National Science Foundation Center for Complex Particle Systems (COMPASS) under award No. 2243104.

## Conflicts of Interest

The authors declare no conflicts of interest.

## Supporting information




**Supporting File**: advs73376‐sup‐0001‐SuppMat.docx.

## Data Availability

All data is available in the manuscript and supporting information.
