# Supplementary material for: Graph‐Theory Approach to Element Miscibility and Alloy Design
Source: Adv Sci (Weinh). 2025 Dec 20;13(16):e21018. doi: 10.1002/advs.202521018 (PMC13042392; doi:10.1002/advs.202521018)
Supplement: Supplementary file 1 — Supporting File: advs73376‐sup‐0001‐SuppMat.docx. [file ADVS-13-e21018-s001.docx]

Supporting Information

Graph-Theory Approach to Element Miscibility and Alloy Design

Andrew Martin,^1*^ Kien Nguyen,^2^ Sebastian Zaatini,^1^ Michael Lastovich, ^1^ Bharat Gwalani,^1^ Paul Bogdan,^2^ Martin M. Thuo^1*^

^1^Department of Materials Science & Engineering, North Carolina State University. Raleigh, NC 27695

^2^Ming Hsieh Department of Electrical and Computer Engineering, University of Southern California. Los Angeles, CA 90089. USA

**Table S1.** Full adjacency matrix of 72 element preferential interactivity parameter (highlights different local groups such as 3d, 4d, 5d transition metals and Lanthanides).


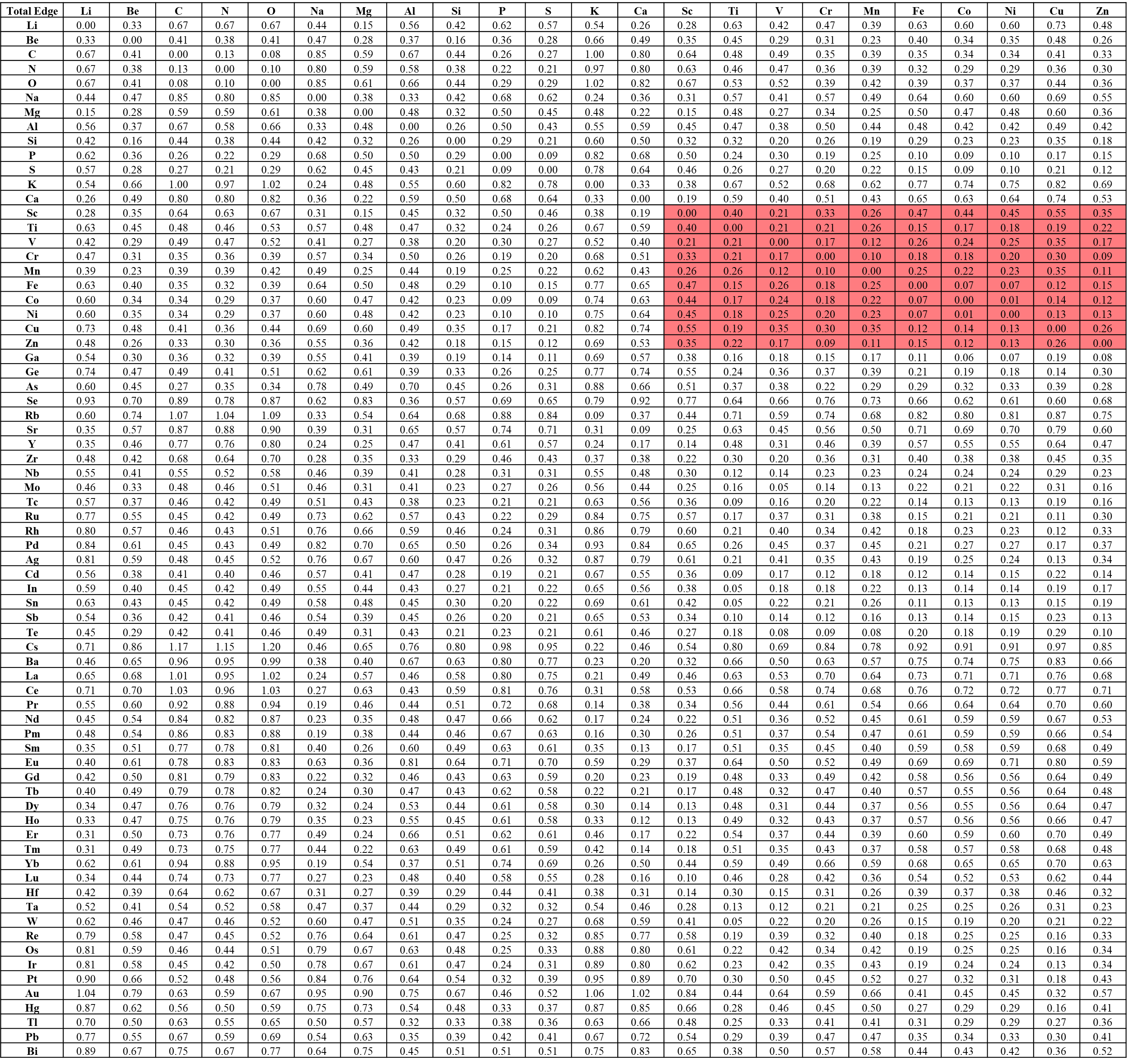


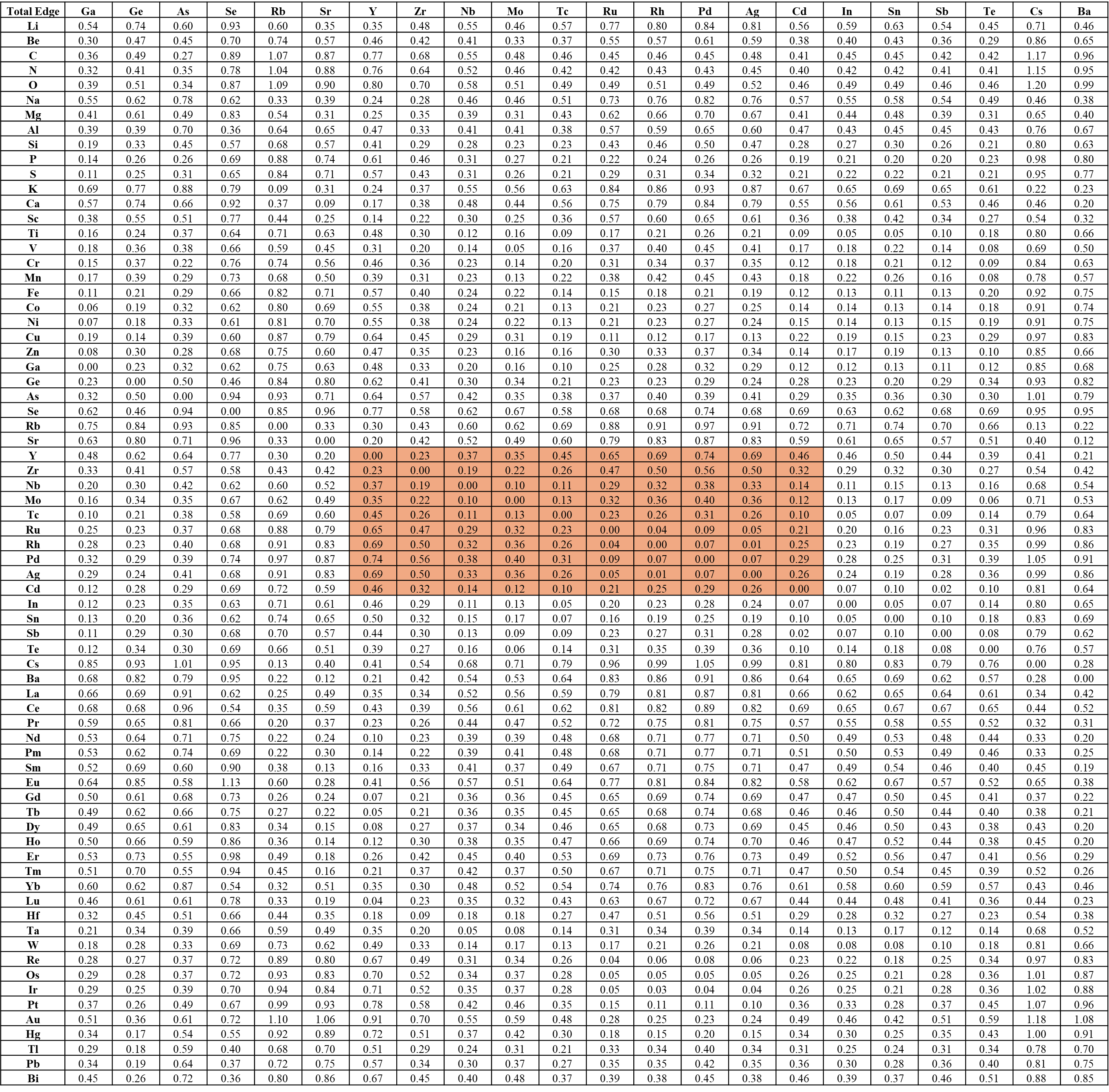


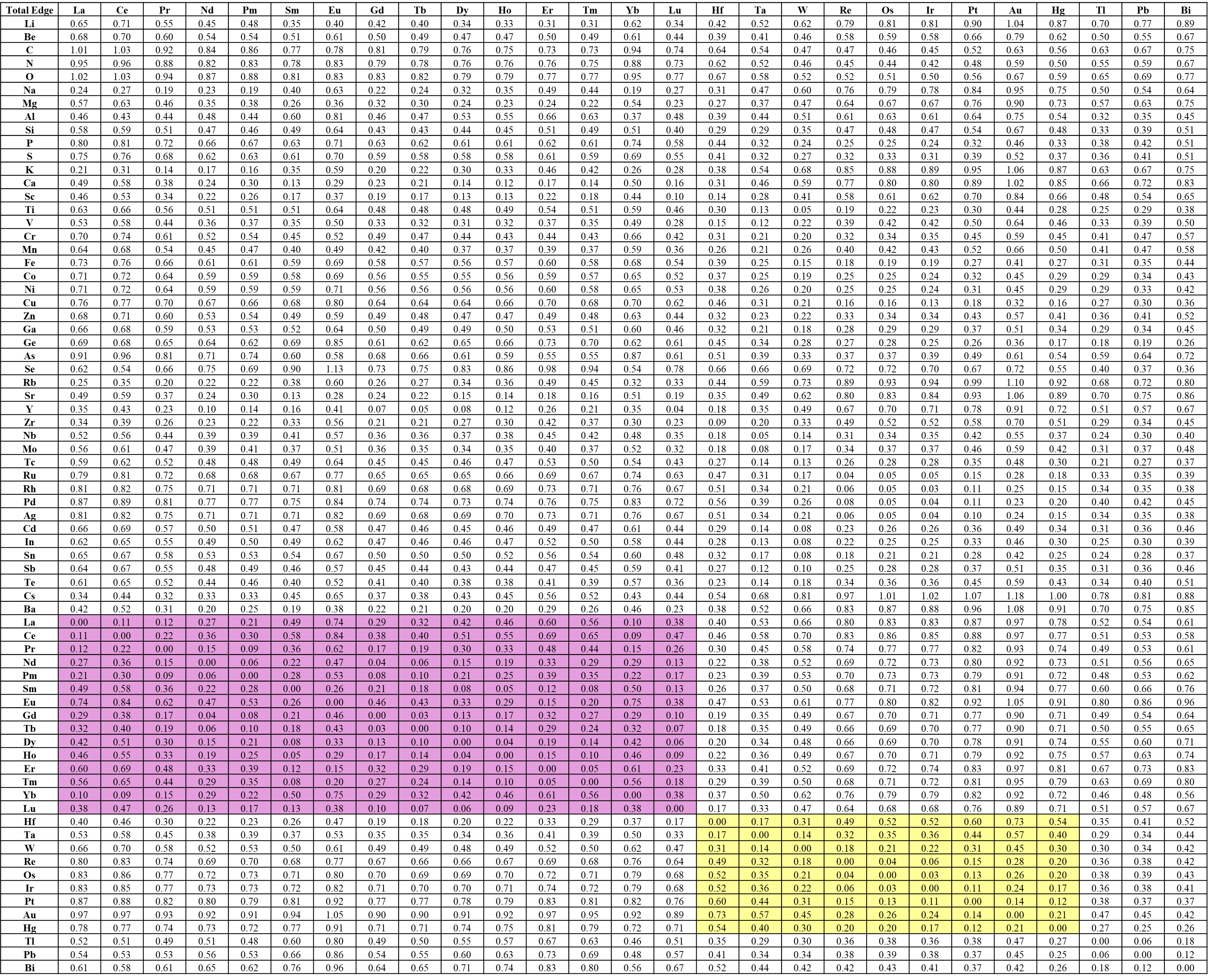


**Table S2.** Closeness centrality values of 72 elements ranked from highest to lowest (Highlight shows average value, 2.22).

| **Atomic #** | **Element** | **Closeness Centrality** |
| --- | --- | --- |
| 42 | Mo | 2.932 |
| 23 | V | 2.904 |
| 43 | Tc | 2.872 |
| 51 | Sb | 2.865 |
| 41 | Nb | 2.864 |
| 52 | Te | 2.863 |
| 73 | Ta | 2.855 |
| 49 | In | 2.851 |
| 48 | Cd | 2.811 |
| 31 | Ga | 2.766 |
| 50 | Sn | 2.748 |
| 22 | Ti | 2.739 |
| 72 | Hf | 2.727 |
| 30 | Zn | 2.676 |
| 74 | W | 2.666 |
| 25 | Mn | 2.662 |
| 40 | Zr | 2.637 |
| 27 | Co | 2.618 |
| 24 | Cr | 2.617 |
| 28 | Ni | 2.594 |
| 26 | Fe | 2.573 |
| 21 | Sc | 2.494 |
| 14 | Si | 2.49 |
| 16 | S | 2.395 |
| 71 | Lu | 2.352 |
| 15 | P | 2.349 |
| 29 | Cu | 2.334 |
| 81 | Tl | 2.322 |
| 39 | Y | 2.291 |
| 65 | Tb | 2.289 |
| 64 | Gd | 2.264 |
| 44 | Ru | 2.262 |
| 66 | Dy | 2.261 |
| 32 | Ge | 2.246 |
| 67 | Ho | 2.212 |
| 12 | Mg | 2.194 |
| 60 | Nd | 2.169 |
| 75 | Re | 2.169 |
| 45 | Rh | 2.157 |
| 61 | Pm | 2.136 |
| 47 | Ag | 2.133 |
| 82 | Pb | 2.127 |
| 62 | Sm | 2.11 |
| 77 | Ir | 2.097 |
| 4 | Be | 2.095 |
| 76 | Os | 2.094 |
| 69 | Tm | 2.055 |
| 13 | Al | 1.991 |
| 46 | Pd | 1.975 |
| 80 | Hg | 1.967 |
| 68 | Er | 1.955 |
| 11 | Na | 1.953 |
| 59 | Pr | 1.949 |
| 20 | Ca | 1.946 |
| 33 | As | 1.917 |
| 78 | Pt | 1.849 |
| 38 | Sr | 1.823 |
| 83 | Bi | 1.808 |
| 70 | Yb | 1.801 |
| 3 | Li | 1.765 |
| 7 | N | 1.744 |
| 56 | Ba | 1.728 |
| 19 | K | 1.722 |
| 57 | La | 1.707 |
| 6 | C | 1.669 |
| 63 | Eu | 1.609 |
| 58 | Ce | 1.599 |
| 8 | O | 1.593 |
| 37 | Rb | 1.584 |
| 79 | Au | 1.511 |
| 34 | Se | 1.413 |
| 55 | Cs | 1.381 |

**Table S3.** Degree centrality values of 72 elements ranked from highest to lowest (Highlight shows average value, 3.0)

| **Atomic #** | **Element** | **Degree Centrality** |
| --- | --- | --- |
| 45 | Rh | 4.634 |
| 27 | Co | 4.535 |
| 47 | Ag | 4.459 |
| 28 | Ni | 4.439 |
| 51 | Sb | 4.356 |
| 49 | In | 4.328 |
| 48 | Cd | 4.284 |
| 77 | Ir | 4.197 |
| 50 | Sn | 4.075 |
| 22 | Ti | 3.975 |
| 76 | Os | 3.973 |
| 44 | Ru | 3.942 |
| 43 | Tc | 3.913 |
| 31 | Ga | 3.861 |
| 42 | Mo | 3.799 |
| 52 | Te | 3.769 |
| 26 | Fe | 3.707 |
| 75 | Re | 3.674 |
| 74 | W | 3.648 |
| 65 | Tb | 3.64 |
| 64 | Gd | 3.588 |
| 41 | Nb | 3.519 |
| 73 | Ta | 3.518 |
| 23 | V | 3.496 |
| 39 | Y | 3.482 |
| 30 | Zn | 3.48 |
| 71 | Lu | 3.474 |
| 66 | Dy | 3.397 |
| 67 | Ho | 3.31 |
| 46 | Pd | 3.272 |
| 24 | Cr | 3.253 |
| 60 | Nd | 3.23 |
| 29 | Cu | 3.209 |
| 25 | Mn | 3.141 |
| 15 | P | 3.119 |
| 16 | S | 3.059 |
| 71 | Hf | 3.028 |
| 61 | Sm | 2.959 |
| 21 | Sc | 2.952 |
| 61 | Pm | 2.868 |
| 40 | Zr | 2.811 |
| 69 | Tm | 2.771 |
| 32 | Ge | 2.655 |
| 14 | Si | 2.641 |
| 81 | Tl | 2.59 |
| 78 | Pt | 2.581 |
| 20 | Ca | 2.532 |
| 68 | Er | 2.512 |
| 80 | Hg | 2.475 |
| 59 | Pr | 2.431 |
| 82 | Pb | 2.4 |
| 12 | Mg | 2.396 |
| 38 | Sr | 2.386 |
| 19 | K | 2.189 |
| 11 | Na | 2.164 |
| 4 | Be | 2.143 |
| 56 | Ba | 2.141 |
| 70 | Yb | 2.114 |
| 33 | As | 2.053 |
| 57 | La | 2.052 |
| 7 | N | 2.018 |
| 37 | Rb | 1.972 |
| 6 | C | 1.959 |
| 13 | Al | 1.949 |
| 83 | Bi | 1.932 |
| 8 | O | 1.872 |
| 3 | Li | 1.858 |
| 58 | Ce | 1.822 |
| 79 | Au | 1.774 |
| 63 | Eu | 1.737 |
| 55 | Cs | 1.566 |
| 34 | Se | 1.381 |

**Table S4.** Clustering coefficient values of 72 elements ranked from highest to lowest (Highlight shows average value, 0.84)

| **Atomic #** | **Element** | **Clustering Coefficient** |
| --- | --- | --- |
| 49 | In | 1.08 |
| 51 | Sb | 1.076 |
| 48 | Cd | 1.065 |
| 50 | Sn | 1.049 |
| 43 | Tc | 1.045 |
| 27 | Co | 1.035 |
| 22 | Ti | 1.031 |
| 31 | Ga | 1.029 |
| 42 | Mo | 1.026 |
| 28 | Ni | 1.022 |
| 52 | Te | 1.02 |
| 26 | Fe | 1.001 |
| 74 | W | 0.993 |
| 23 | V | 0.988 |
| 41 | Nb | 0.988 |
| 73 | Ta | 0.986 |
| 30 | Zn | 0.975 |
| 45 | Rh | 0.967 |
| 44 | Ru | 0.962 |
| 47 | Ag | 0.951 |
| 24 | Cr | 0.942 |
| 77 | Ir | 0.939 |
| 25 | Mn | 0.927 |
| 76 | Os | 0.924 |
| 75 | Re | 0.919 |
| 29 | Cu | 0.919 |
| 72 | Hf | 0.914 |
| 71 | Lu | 0.908 |
| 15 | P | 0.905 |
| 65 | Tb | 0.904 |
| 39 | Y | 0.898 |
| 16 | S | 0.897 |
| 64 | Gd | 0.892 |
| 66 | Dy | 0.888 |
| 21 | Sc | 0.882 |
| 40 | Zr | 0.877 |
| 67 | Ho | 0.871 |
| 60 | Nd | 0.855 |
| 46 | Pd | 0.855 |
| 14 | Si | 0.847 |
| 32 | Ge | 0.834 |
| 62 | Sm | 0.827 |
| 61 | Pm | 0.822 |
| 81 | Tl | 0.819 |
| 69 | Tm | 0.797 |
| 12 | Mg | 0.783 |
| 80 | Hg | 0.78 |
| 78 | Pt | 0.776 |
| 82 | Pb | 0.772 |
| 20 | Ca | 0.768 |
| 68 | Er | 0.757 |
| 59 | Pr | 0.752 |
| 4 | Be | 0.741 |
| 38 | Sr | 0.736 |
| 11 | Na | 0.724 |
| 33 | As | 0.719 |
| 13 | Al | 0.7 |
| 56 | Ba | 0.698 |
| 19 | K | 0.697 |
| 70 | Yb | 0.691 |
| 7 | N | 0.687 |
| 83 | Bi | 0.681 |
| 57 | La | 0.673 |
| 6 | C | 0.667 |
| 3 | Li | 0.665 |
| 37 | Rb | 0.652 |
| 8 | O | 0.643 |
| 79 | Au | 0.633 |
| 63 | Eu | 0.631 |
| 58 | Ce | 0.63 |
| 55 | Cs | 0.577 |
| 34 | Se | 0.557 |

**Table S5.** Calculated network metrics of 72 elements ordered by atomic number.

| **Atomic #** | **Element** | **Closeness Centrality** | **Degree Centrality** | **Clustering Coefficient** |
| --- | --- | --- | --- | --- |
| 3 | Li | 1.765 | 1.858 | 0.665 |
| 4 | Be | 2.095 | 2.143 | 0.741 |
| 6 | C | 1.669 | 1.959 | 0.667 |
| 7 | N | 1.744 | 2.018 | 0.687 |
| 8 | O | 1.593 | 1.872 | 0.643 |
| 11 | Na | 1.953 | 2.164 | 0.724 |
| 12 | Mg | 2.194 | 2.396 | 0.783 |
| 13 | Al | 1.991 | 1.949 | 0.7 |
| 14 | Si | 2.49 | 2.641 | 0.847 |
| 15 | P | 2.349 | 3.119 | 0.905 |
| 16 | S | 2.395 | 3.059 | 0.897 |
| 19 | K | 1.722 | 2.189 | 0.697 |
| 20 | Ca | 1.946 | 2.532 | 0.768 |
| 21 | Sc | 2.494 | 2.952 | 0.882 |
| 22 | Ti | 2.739 | 3.975 | 1.031 |
| 23 | V | 2.904 | 3.496 | 0.988 |
| 24 | Cr | 2.617 | 3.253 | 0.942 |
| 25 | Mn | 2.662 | 3.141 | 0.927 |
| 26 | Fe | 2.573 | 3.707 | 1.001 |
| 27 | Co | 2.618 | 4.535 | 1.035 |
| 28 | Ni | 2.594 | 4.439 | 1.022 |
| 29 | Cu | 2.334 | 3.209 | 0.919 |
| 30 | Zn | 2.676 | 3.48 | 0.975 |
| 31 | Ga | 2.766 | 3.861 | 1.029 |
| 32 | Ge | 2.246 | 2.655 | 0.834 |
| 33 | As | 1.917 | 2.053 | 0.719 |
| 34 | Se | 1.413 | 1.381 | 0.557 |
| 37 | Rb | 1.584 | 1.972 | 0.652 |
| 38 | Sr | 1.823 | 2.386 | 0.736 |
| 39 | Y | 2.291 | 3.482 | 0.898 |
| 40 | Zr | 2.637 | 2.811 | 0.877 |
| 41 | Nb | 2.864 | 3.519 | 0.988 |
| 42 | Mo | 2.932 | 3.799 | 1.026 |
| 43 | Tc | 2.872 | 3.913 | 1.045 |
| 44 | Ru | 2.262 | 3.942 | 0.962 |
| 45 | Rh | 2.157 | 4.634 | 0.967 |
| 46 | Pd | 1.975 | 3.272 | 0.855 |
| 47 | Ag | 2.133 | 4.459 | 0.951 |
| 48 | Cd | 2.811 | 4.284 | 1.065 |
| 49 | In | 2.851 | 4.328 | 1.08 |
| 50 | Sn | 2.748 | 4.075 | 1.049 |
| 51 | Sb | 2.865 | 4.356 | 1.076 |
| 52 | Te | 2.863 | 3.769 | 1.02 |
| 55 | Cs | 1.381 | 1.566 | 0.577 |
| 56 | Ba | 1.728 | 2.141 | 0.698 |
| 57 | La | 1.707 | 2.052 | 0.673 |
| 58 | Ce | 1.599 | 1.822 | 0.63 |
| 59 | Pr | 1.949 | 2.431 | 0.752 |
| 60 | Nd | 2.169 | 3.23 | 0.855 |
| 61 | Pm | 2.136 | 2.959 | 0.822 |
| 62 | Sm | 2.11 | 2.868 | 0.827 |
| 63 | Eu | 1.609 | 1.737 | 0.631 |
| 64 | Gd | 2.264 | 3.588 | 0.892 |
| 65 | Tb | 2.289 | 3.64 | 0.904 |
| 66 | Dy | 2.261 | 3.397 | 0.888 |
| 67 | Ho | 2.212 | 3.31 | 0.871 |
| 68 | Er | 1.955 | 2.512 | 0.757 |
| 69 | Tm | 2.055 | 2.771 | 0.797 |
| 70 | Yb | 1.801 | 2.114 | 0.691 |
| 71 | Lu | 2.352 | 3.474 | 0.908 |
| 72 | Hf | 2.727 | 3.028 | 0.914 |
| 73 | Ta | 2.855 | 3.518 | 0.986 |
| 74 | W | 2.666 | 3.648 | 0.993 |
| 75 | Re | 2.169 | 3.674 | 0.919 |
| 76 | Os | 2.094 | 3.973 | 0.924 |
| 77 | Ir | 2.097 | 4.197 | 0.939 |
| 78 | Pt | 1.849 | 2.581 | 0.776 |
| 79 | Au | 1.511 | 1.774 | 0.633 |
| 80 | Hg | 1.967 | 2.475 | 0.78 |
| 81 | Tl | 2.322 | 2.59 | 0.819 |
| 82 | Pb | 2.127 | 2.4 | 0.772 |
| 83 | Bi | 1.808 | 1.932 | 0.681 |

**Table S6.** Normalized covalent radius, CED and E^0^ values of 72 elements used in this work

| **Atomic #** | **Element** | **Normalized Radius** | **Normalized CED** | **Normalized E0** |
| --- | --- | --- | --- | --- |
| 3 | Li | 0.37931 | 0.783786 | 0 |
| 4 | Be | 0.201149 | 0.650268 | 0.244969 |
| 6 | C | 0.063218 | 0.845727 | 0.583162 |
| 7 | N | 0.04023 | 0.722712 | 0.577002 |
| 8 | O | 0 | 0.814541 | 0.556468 |
| 11 | Na | 0.551724 | 0.384068 | 0.067762 |
| 12 | Mg | 0.436782 | 0.743277 | 0.137166 |
| 13 | Al | 0.310345 | 0.300109 | 0.283368 |
| 14 | Si | 0.287356 | 0.550475 | 0.342916 |
| 15 | P | 0.258621 | 0.682293 | 0.601437 |
| 16 | S | 0.229885 | 0.639858 | 0.526433 |
| 19 | K | 0.781609 | 0.430739 | 0.022382 |
| 20 | Ca | 0.632184 | 0.728419 | 0.035318 |
| 21 | Sc | 0.545977 | 0.668492 | 0.197125 |
| 22 | Ti | 0.482759 | 0.606804 | 0.59076 |
| 23 | V | 0.45977 | 0.637105 | 0.38193 |
| 24 | Cr | 0.37931 | 0.753332 | 0.472279 |
| 25 | Mn | 0.373563 | 0.720152 | 0.380903 |
| 26 | Fe | 0.344828 | 0.643986 | 0.616016 |
| 27 | Co | 0.310345 | 0.611474 | 0.566735 |
| 28 | Ni | 0.304598 | 0.600466 | 0.570842 |
| 29 | Cu | 0.333333 | 0.559835 | 0.694045 |
| 30 | Zn | 0.321839 | 0.679725 | 0.468172 |
| 31 | Ga | 0.33908 | 0.61841 | 0.511499 |
| 32 | Ge | 0.321839 | 0.434191 | 0.644764 |
| 33 | As | 0.321839 | 0.934056 | 0.575359 |
| 34 | Se | 0.310345 | 0 | 0.486653 |
| 37 | Rb | 0.867816 | 0.438857 | 0.01232 |
| 38 | Sr | 0.724138 | 0.736673 | 0.028953 |
| 39 | Y | 0.643678 | 0.594384 | 0.135524 |
| 40 | Zr | 0.574713 | 0.487374 | 0.326489 |
| 41 | Nb | 0.528736 | 0.577947 | 0.482546 |
| 42 | Mo | 0.471264 | 0.648233 | 0.434086 |
| 43 | Tc | 0.425287 | 0.569043 | 0.526899 |
| 44 | Ru | 0.413793 | 0.621528 | 0.747433 |
| 45 | Rh | 0.402299 | 0.602178 | 0.780287 |
| 46 | Pd | 0.37931 | 0.648244 | 0.827515 |
| 47 | Ag | 0.413793 | 0.598082 | 0.788501 |
| 48 | Cd | 0.436782 | 0.672196 | 0.542094 |
| 49 | In | 0.448276 | 0.608753 | 0.554825 |
| 50 | Sn | 0.436782 | 0.593055 | 0.595483 |
| 51 | Sb | 0.436782 | 0.663034 | 0.519507 |
| 52 | Te | 0.41954 | 0.681218 | 0.439425 |
| 55 | Cs | 1 | 0.447646 | 0.002875 |
| 56 | Ba | 0.816092 | 0.65518 | 0.026283 |
| 57 | La | 0.747126 | 0.259928 | 0.135524 |
| 58 | Ce | 0.689655 | 0.163045 | 0.144559 |
| 59 | Pr | 0.724138 | 0.379704 | 0.141068 |
| 60 | Nd | 0.712644 | 0.527303 | 0.147228 |
| 61 | Pm | 0.701149 | 0.466046 | 0.151951 |
| 62 | Sm | 0.695402 | 0.743359 | 0.151129 |
| 63 | Eu | 0.683908 | 1 | 0.129979 |
| 64 | Gd | 0.678161 | 0.538398 | 0.156263 |
| 65 | Tb | 0.672414 | 0.566093 | 0.156057 |
| 66 | Dy | 0.666667 | 0.669584 | 0.152977 |
| 67 | Ho | 0.66092 | 0.707708 | 0.145791 |
| 68 | Er | 0.649425 | 0.854011 | 0.145585 |
| 69 | Tm | 0.649425 | 0.808529 | 0.148049 |
| 70 | Yb | 0.655172 | 0.24776 | 0.156057 |
| 71 | Lu | 0.632184 | 0.626418 | 0.156057 |
| 72 | Hf | 0.574713 | 0.579627 | 0.305955 |
| 73 | Ta | 0.54023 | 0.622314 | 0.469815 |
| 74 | W | 0.494253 | 0.653851 | 0.599795 |
| 75 | Re | 0.442529 | 0.645193 | 0.772074 |
| 76 | Os | 0.413793 | 0.645403 | 0.795483 |
| 77 | Ir | 0.390805 | 0.623902 | 0.795688 |
| 78 | Pt | 0.37931 | 0.547826 | 0.870637 |
| 79 | Au | 0.37931 | 0.494312 | 1 |
| 80 | Hg | 0.390805 | 0.449881 | 0.798768 |
| 81 | Tl | 0.45977 | 0.360611 | 0.554415 |
| 82 | Pb | 0.465517 | 0.313044 | 0.597536 |
| 83 | Bi | 0.494253 | 0.240746 | 0.689322 |


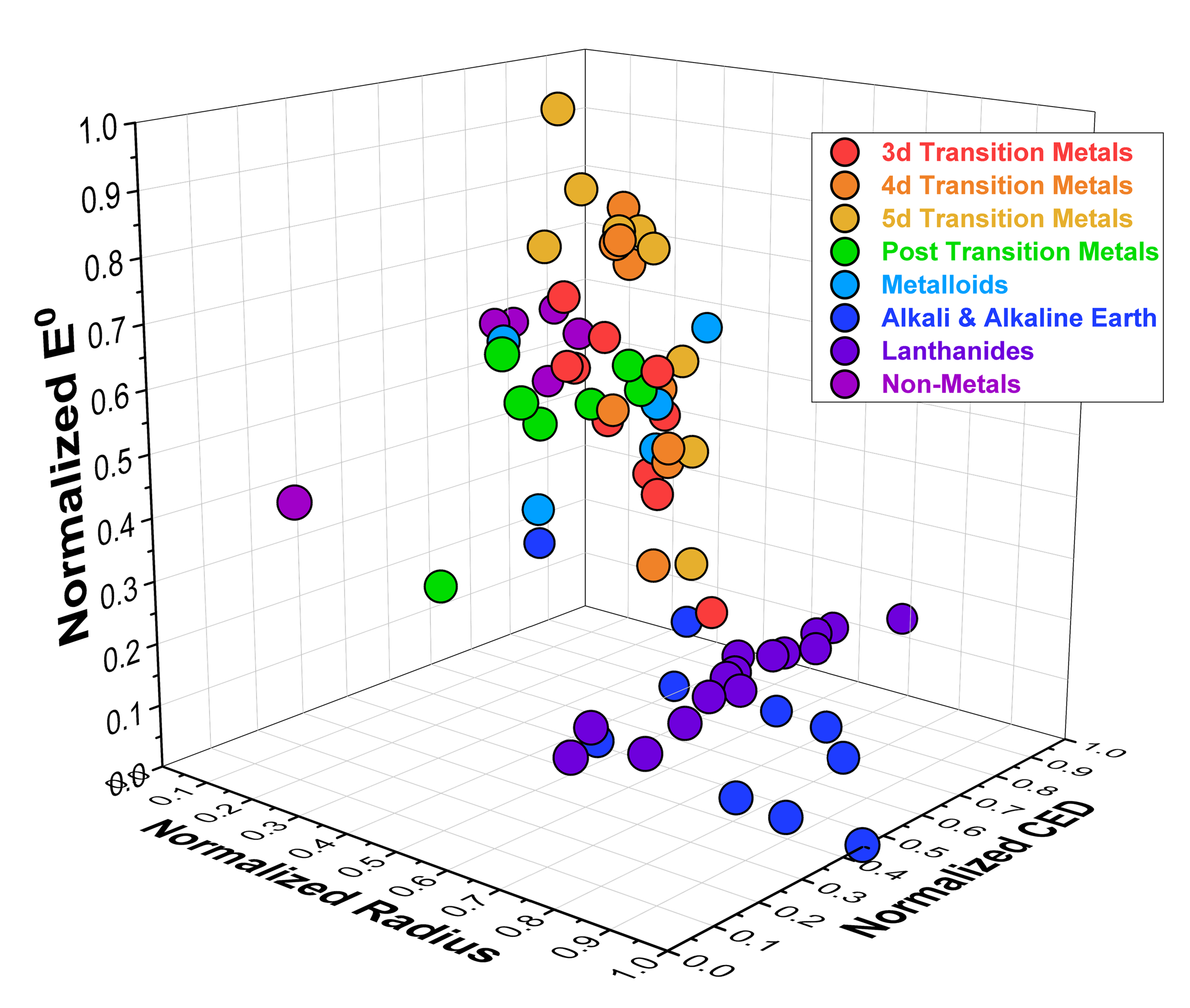


**Figure S1.** Preferential Interactivity Parameter of 72 elements differentiated by periodic key groups.


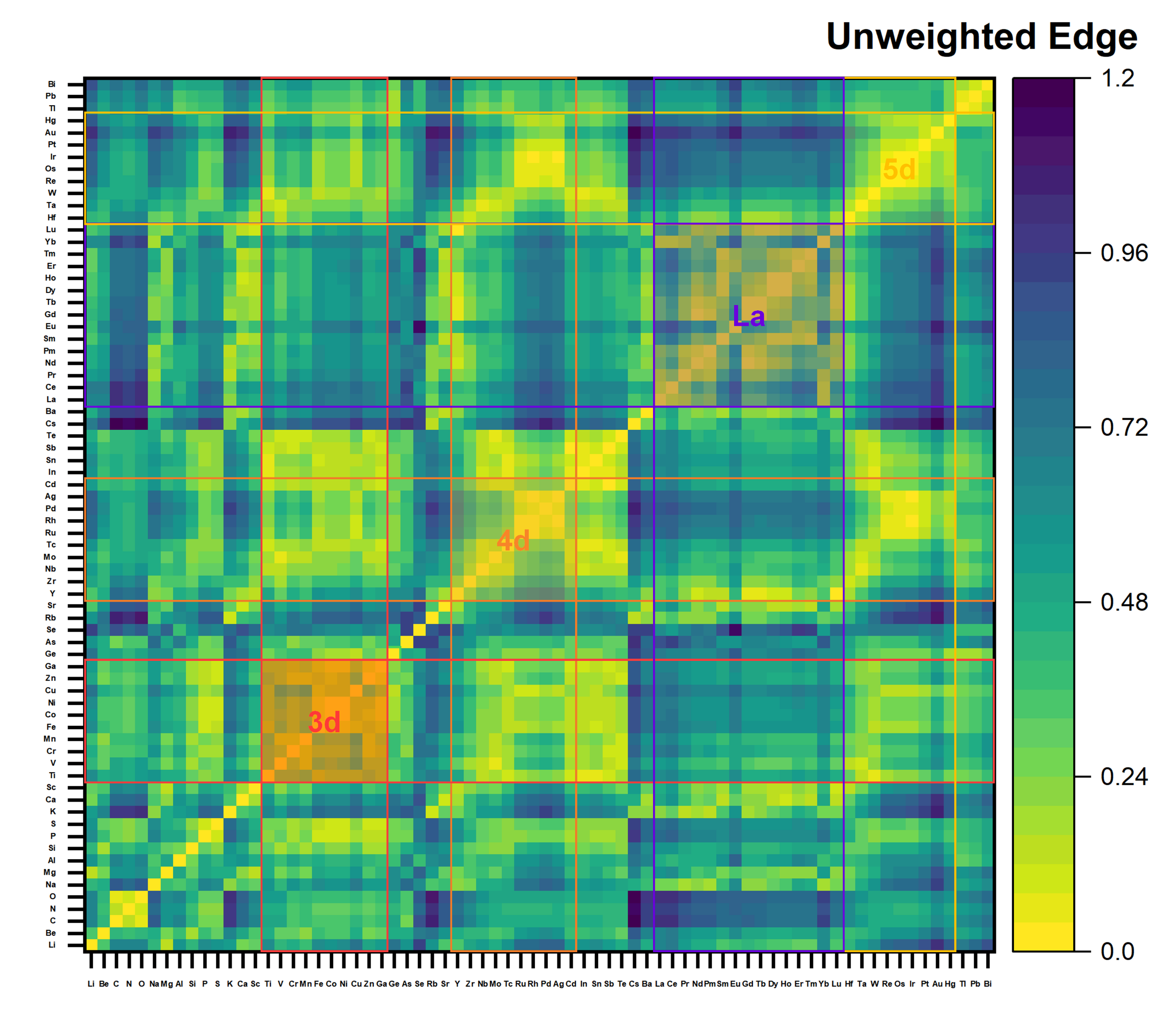


**Figure S2.** Full scale image of Preferential Interactivity Parameter heatmap for 72 elements with line guides indicating group intersections.


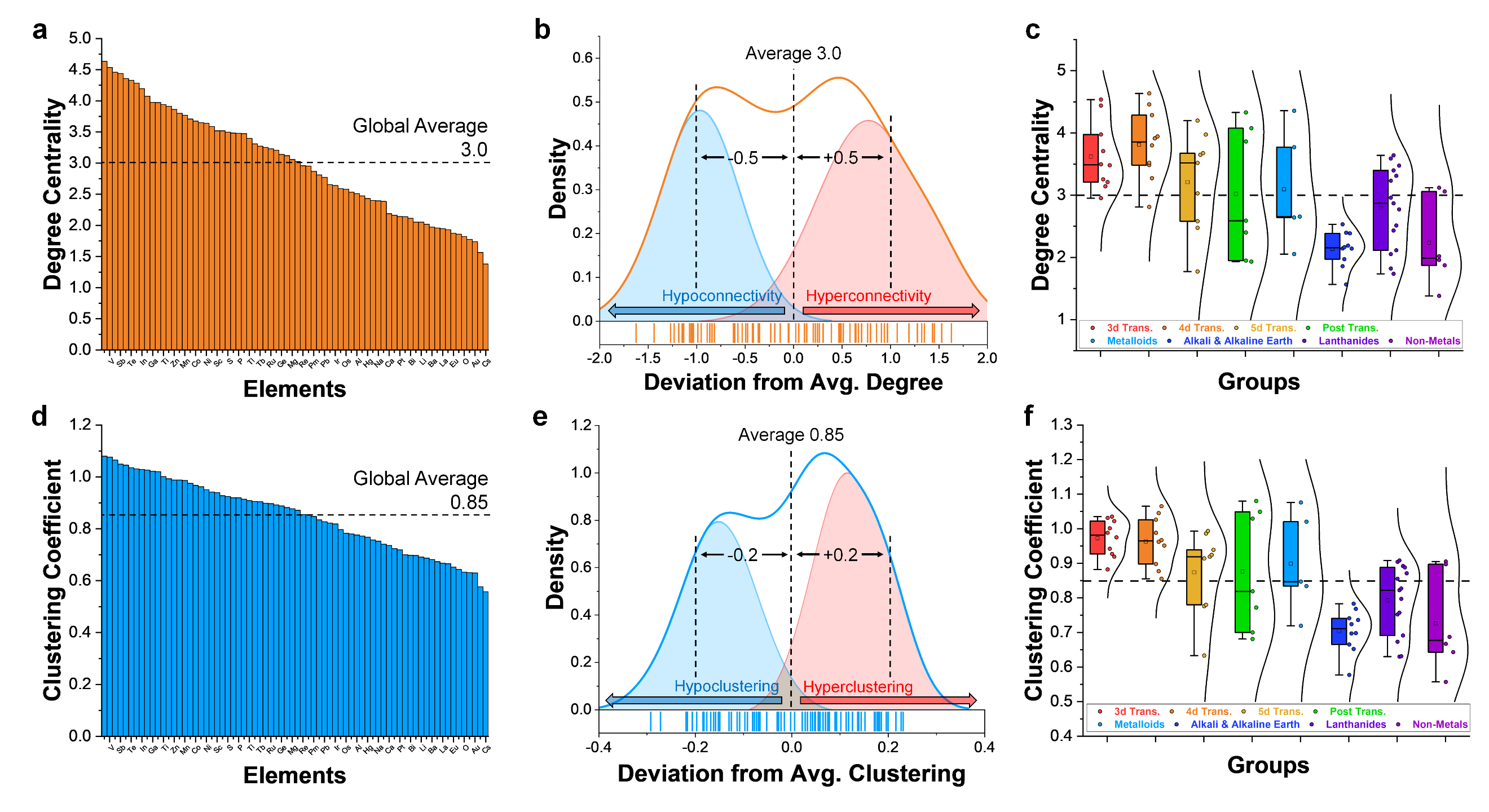


**Figure S3.** (a) Global degree centrality values of 72 elements analyzed in this work. (b) Bimodal distribution of closeness centrality standard deviation indicating distinct groupings of hypo-connectivity, hyper-connectivity elements. (c) Statistical analysis of different elemental group’s degree centrality values. (d) Global clustering coefficient values of 72 elements analyzed in this work. (e) Bimodal distribution of clustering coefficient standard deviation indicating distinct groupings of hypo-clustering, hyper-clustering elements. (f) Statistical analysis of different elemental group’s clustering coefficient values.


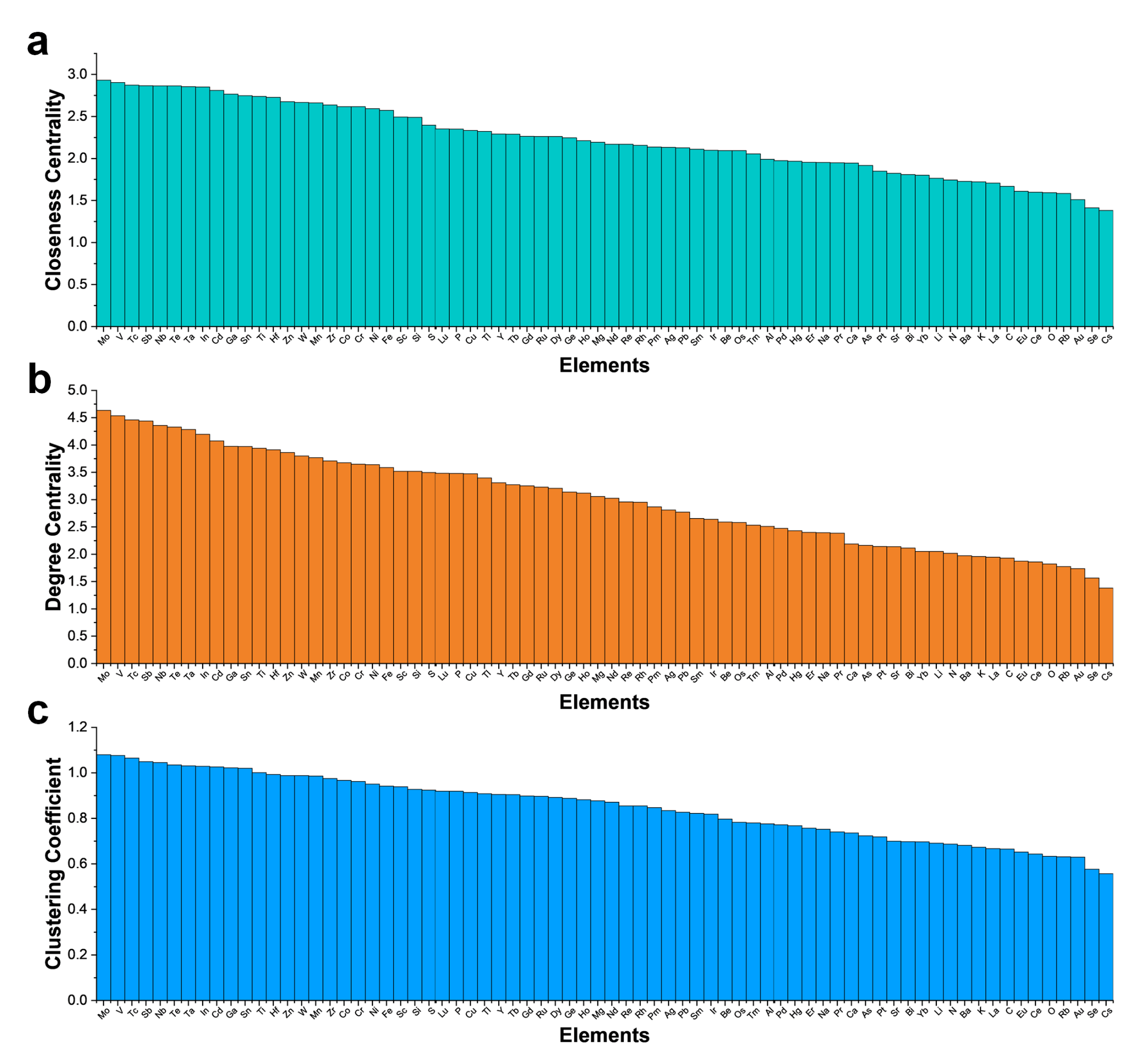


**Figure S4.** (a) Full scale global closeness centrality values of 72 elements analyzed in this work. (b) Full scale global degree centrality values of 72 elements analyzed in this work. (c) Full scale global clustering coefficient values of 72 elements analyzed in this work.
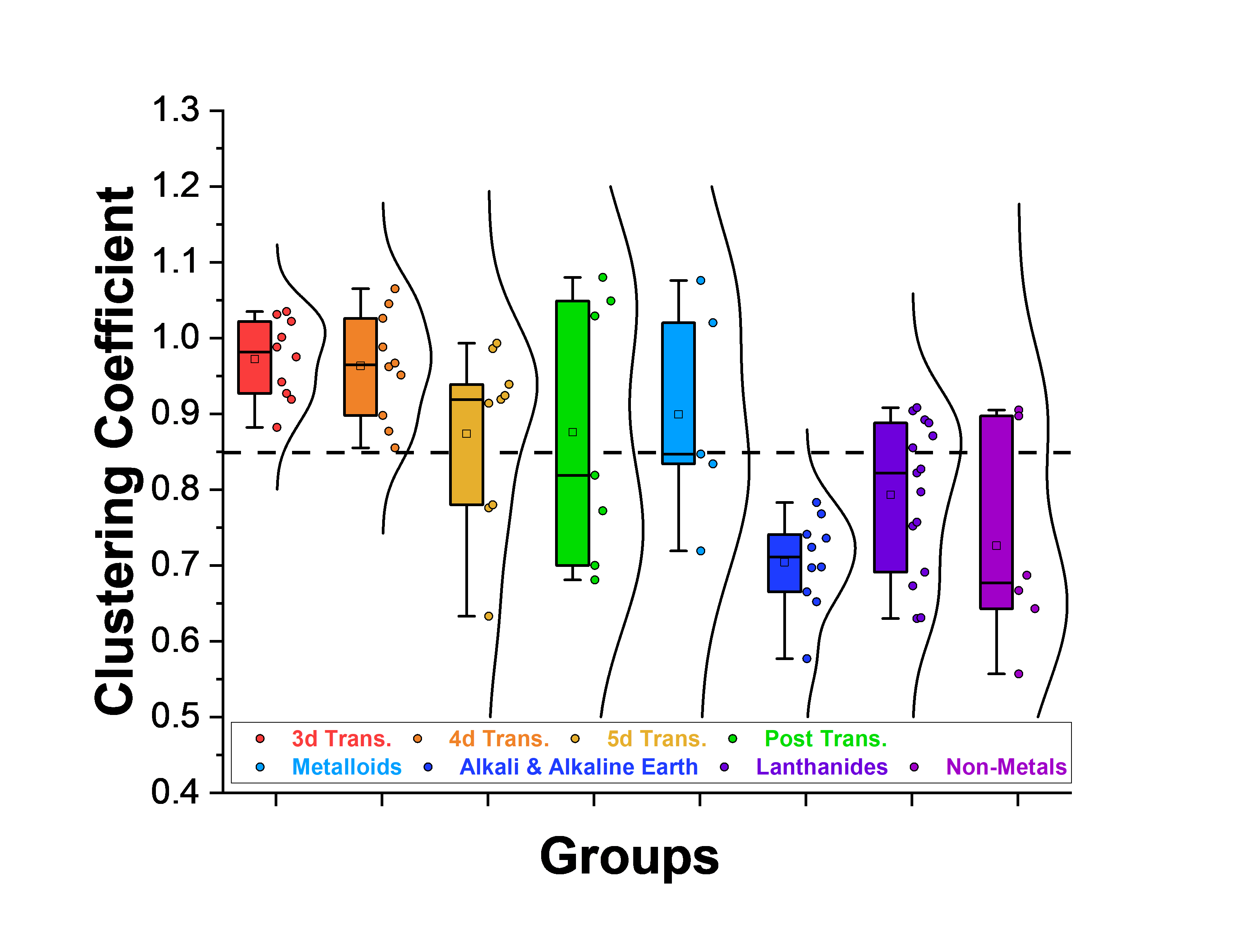

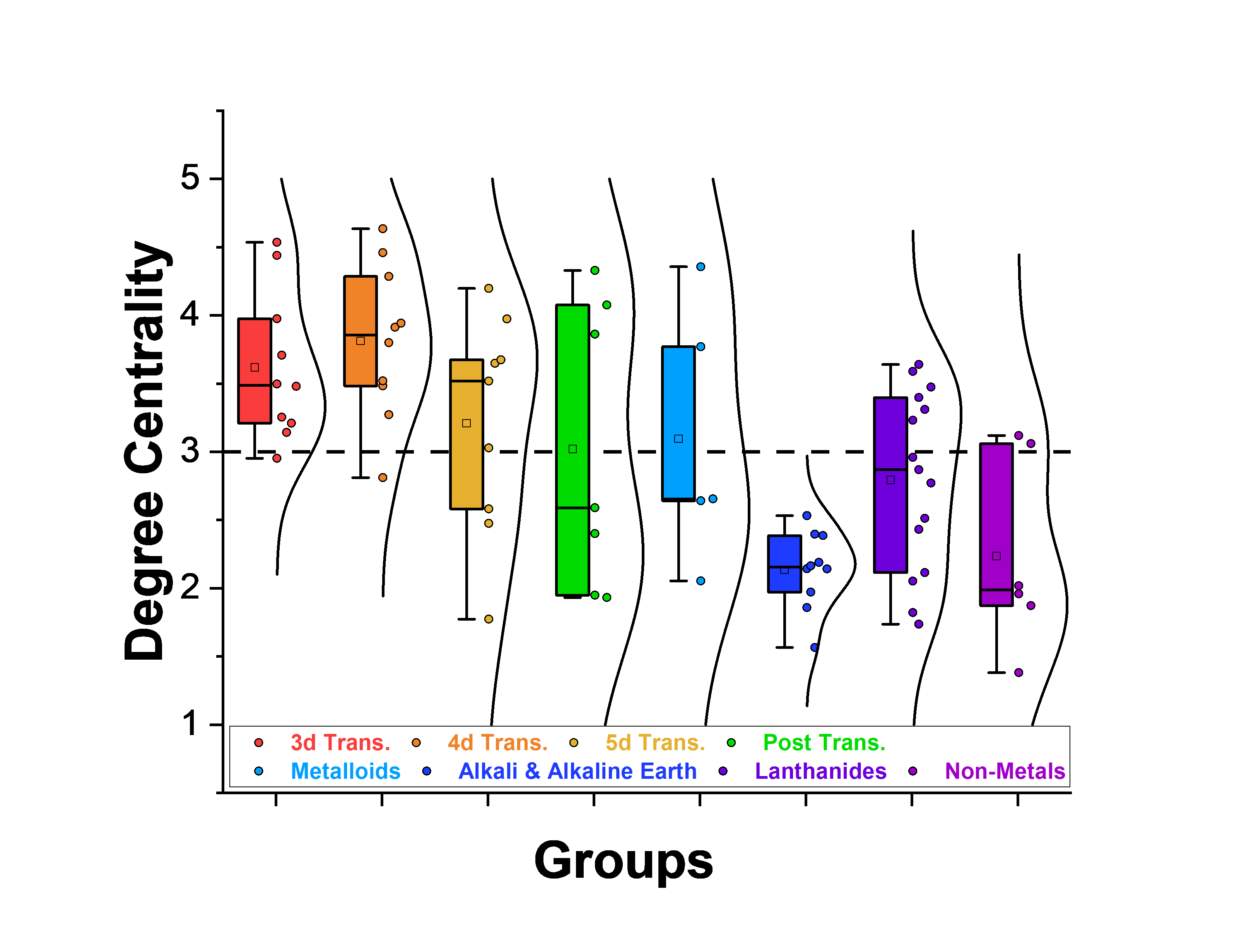

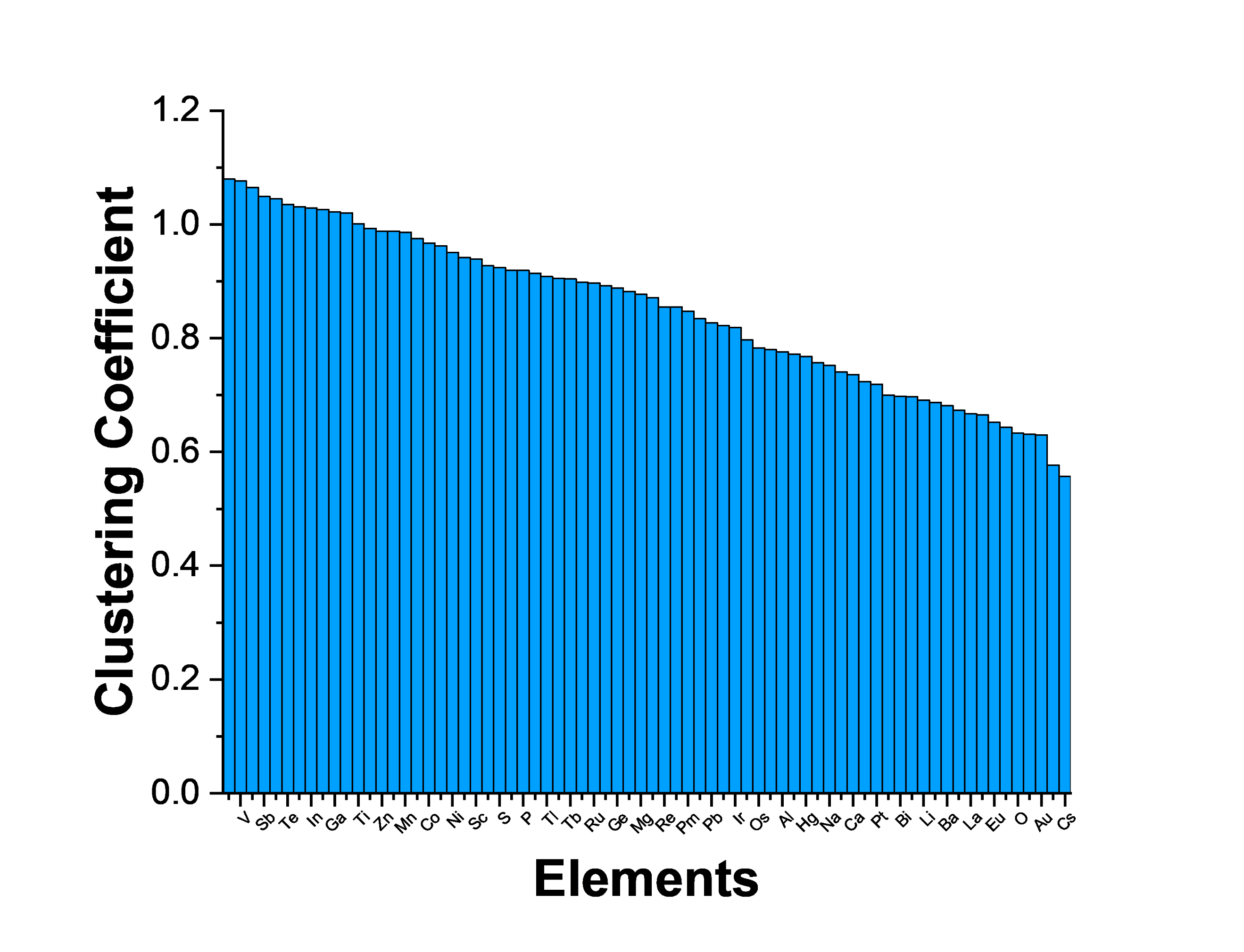


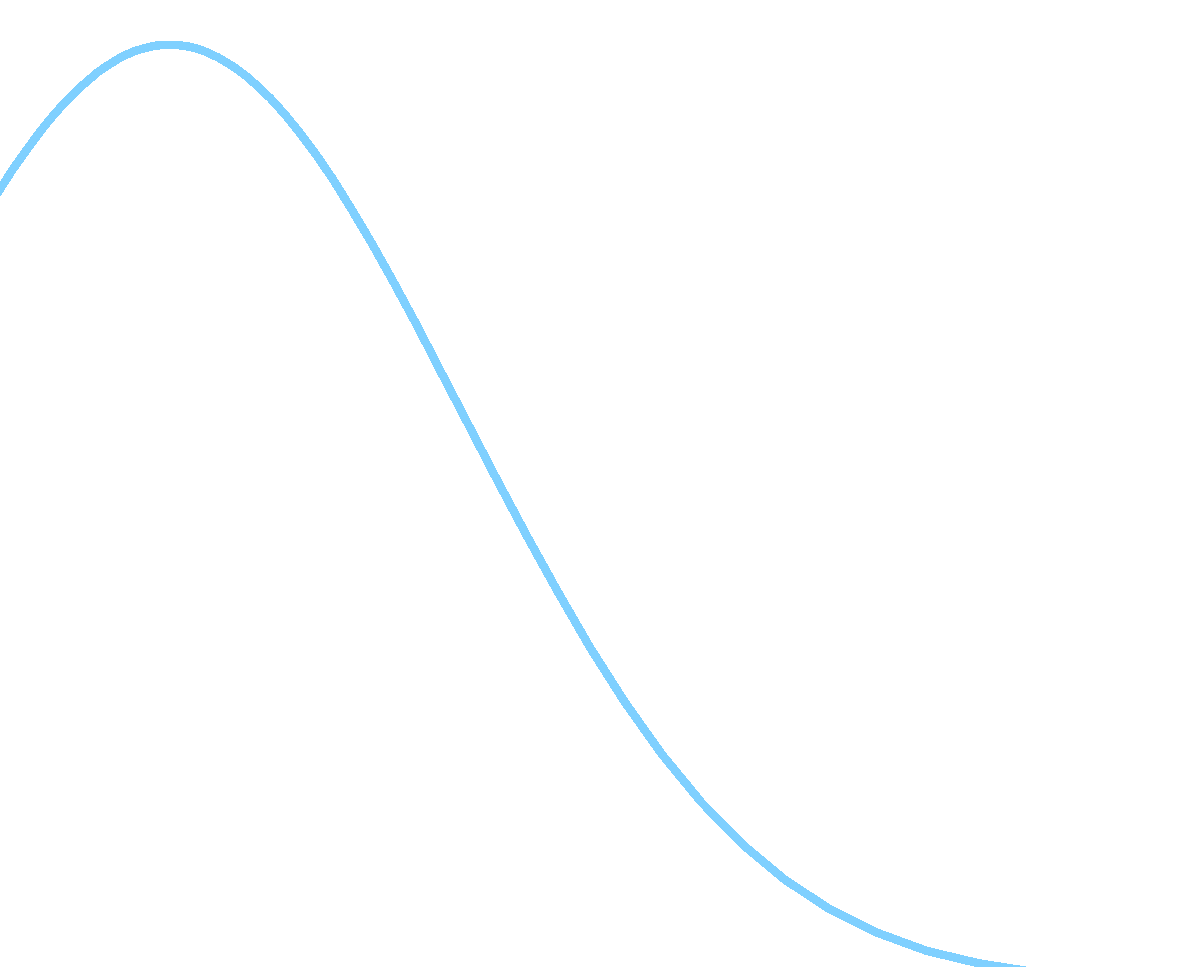

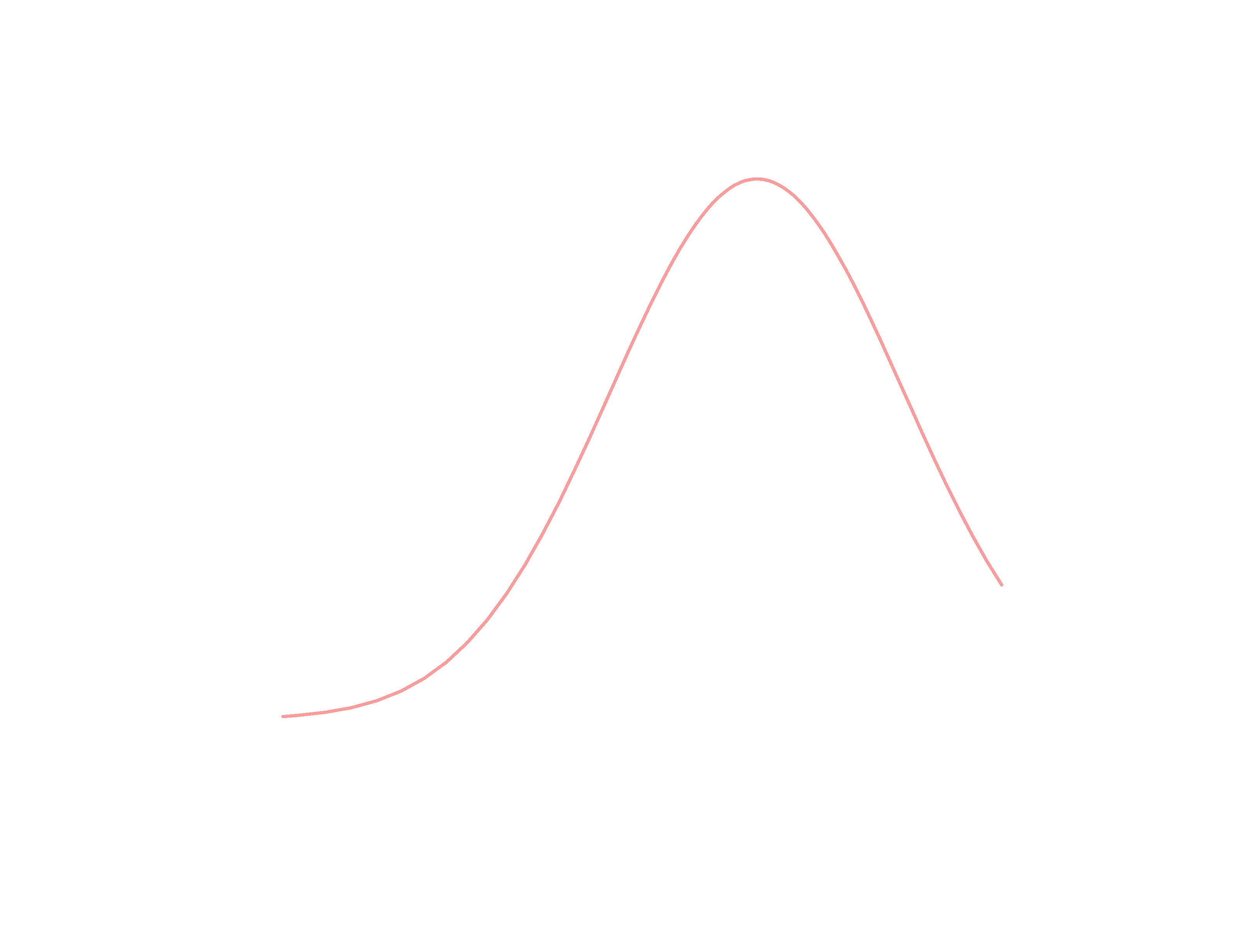

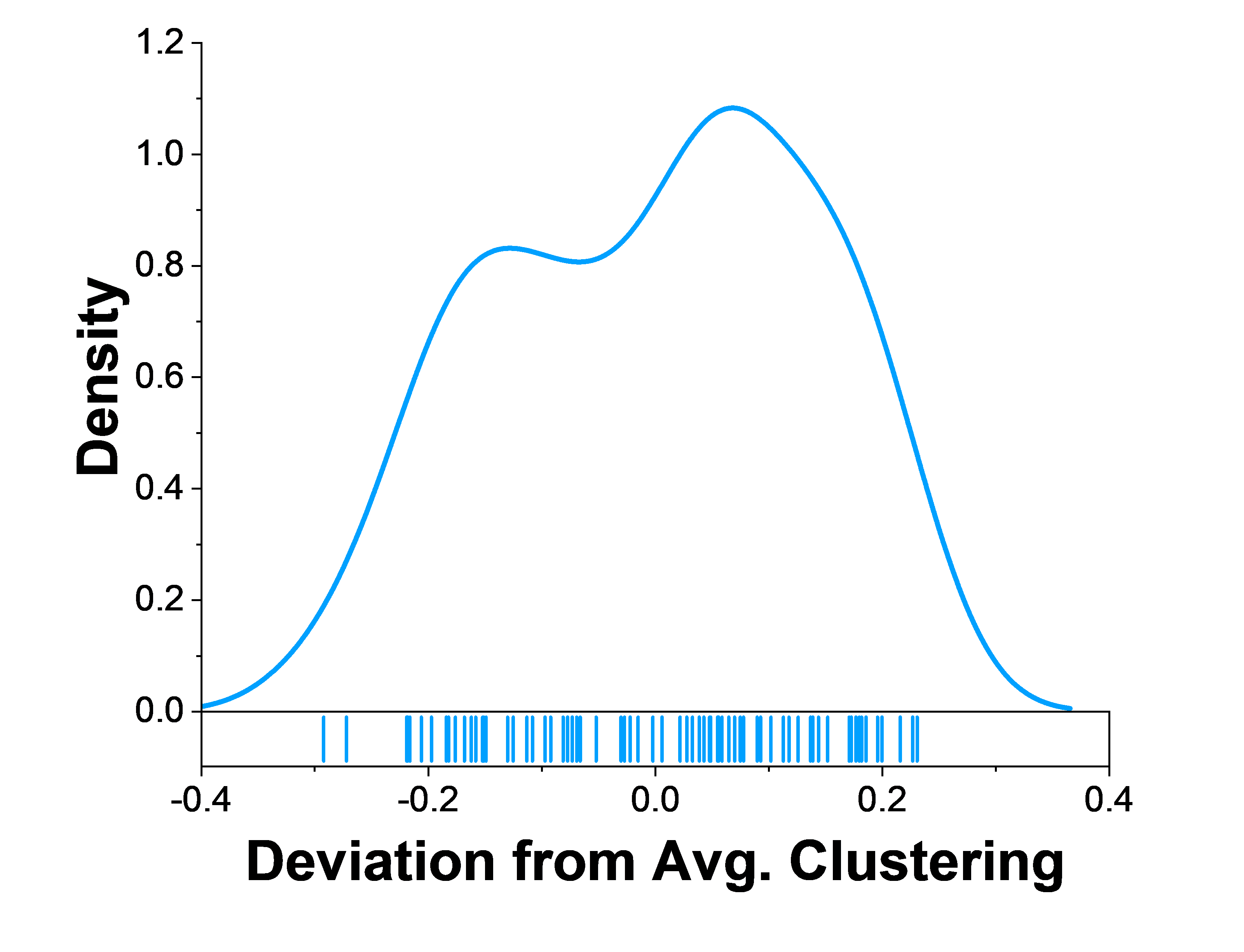


Hypoclustering

Hyperclustering

Average 0.85

-0.2

+0.2

Global Average

3.0

Global Average

0.85

**b**

**c**

**e**

**f**

**d**


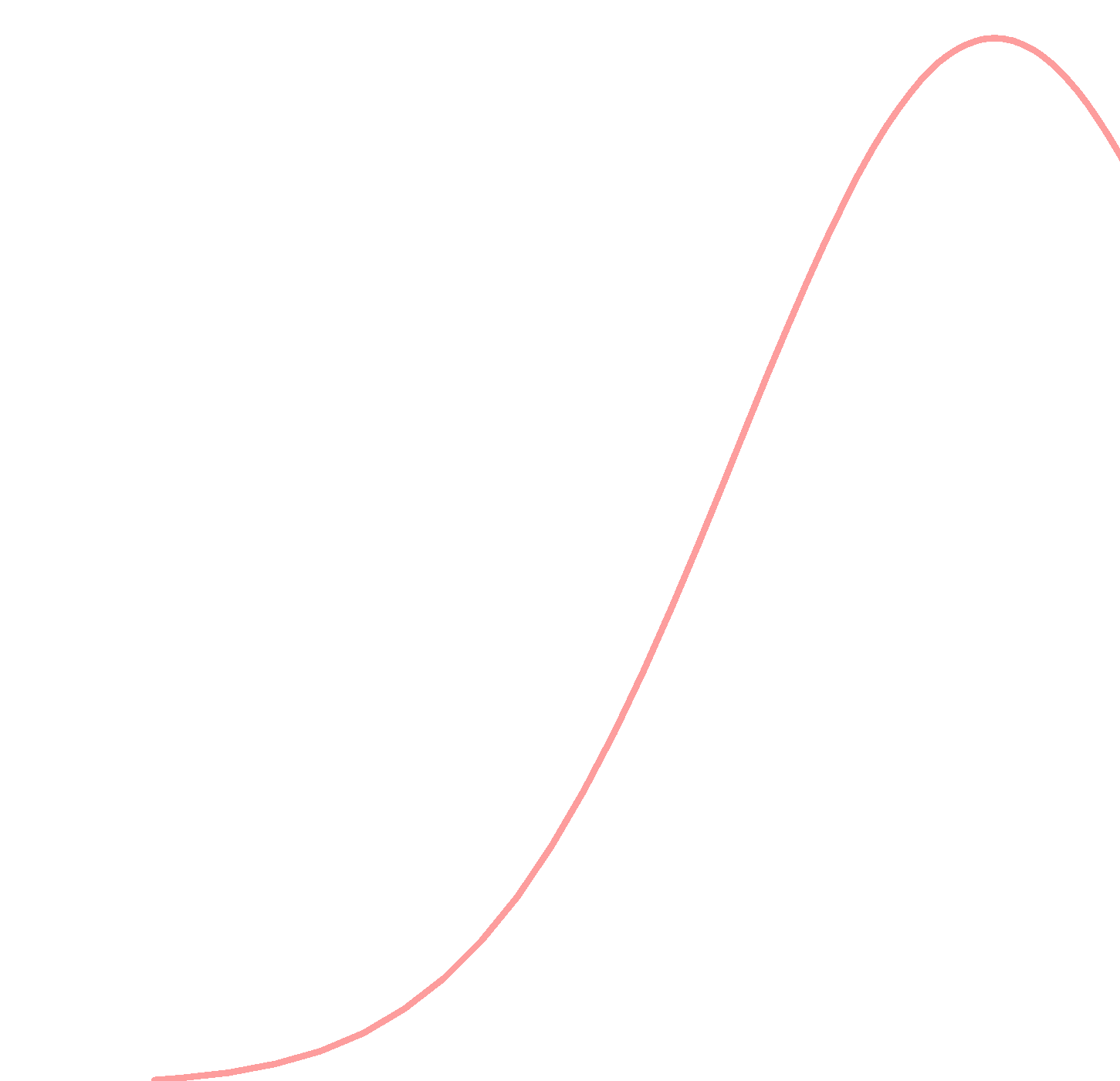

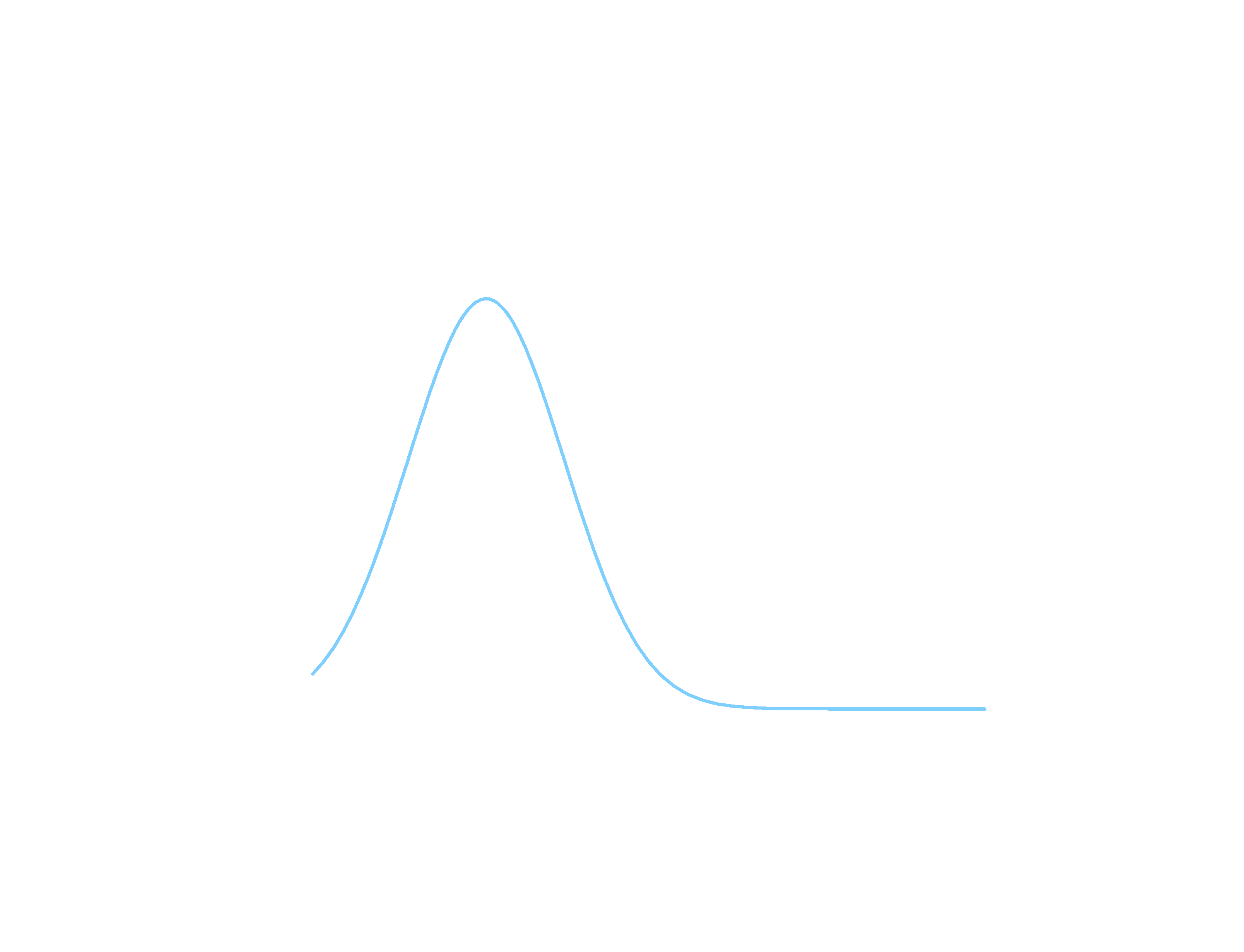

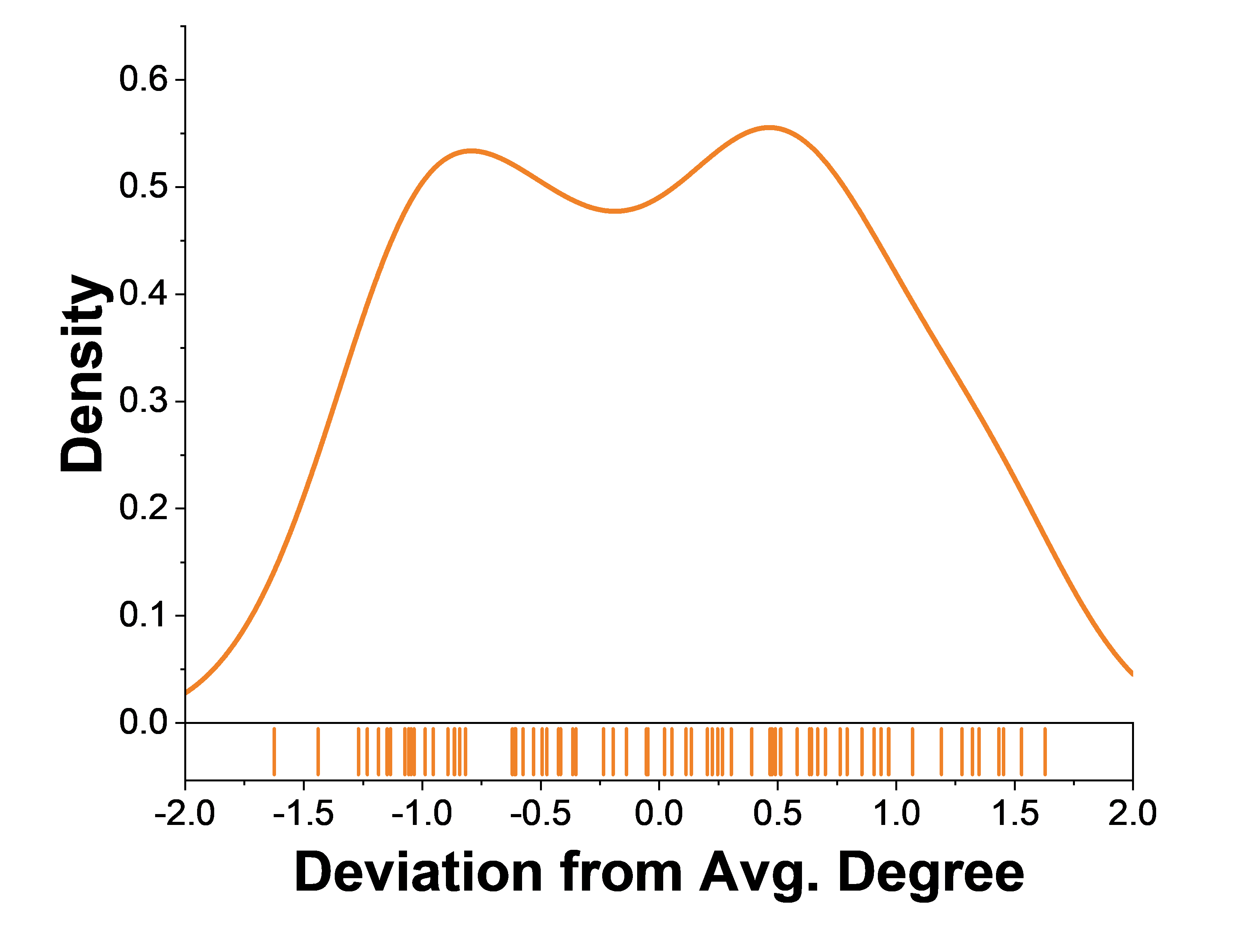


Hypoconnectivity

Hyperconnectivity

Average 3.0

-0.5

+0.5


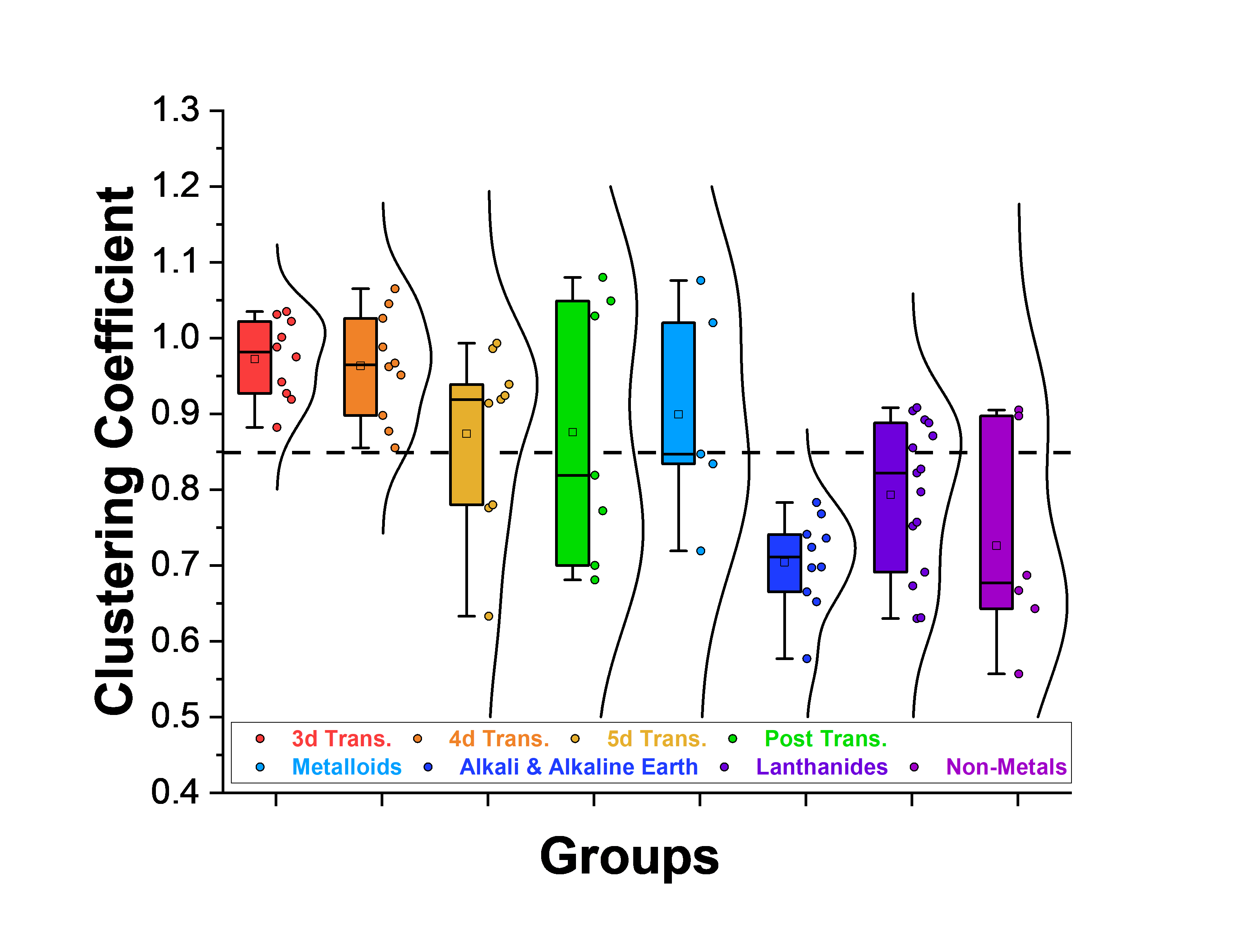

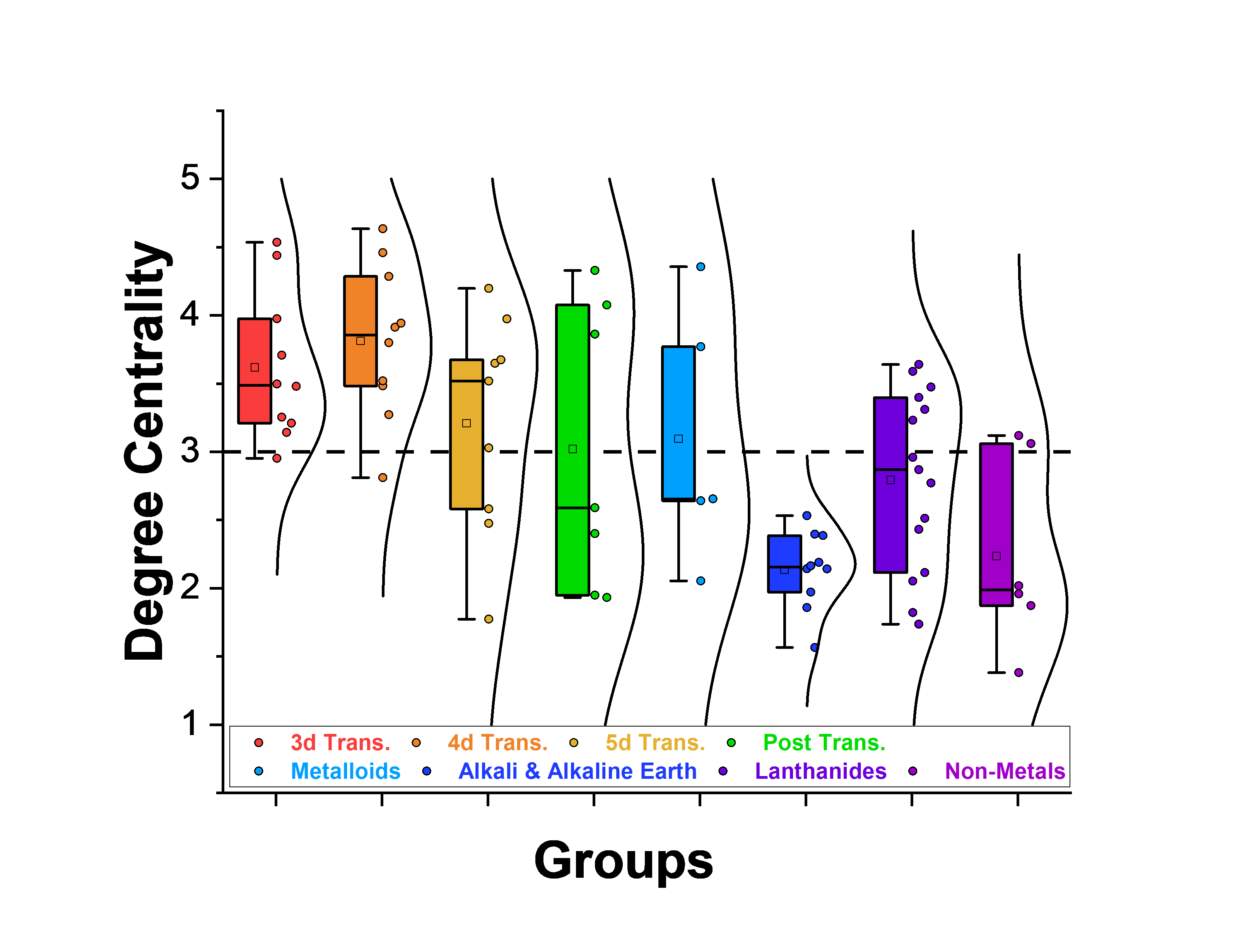

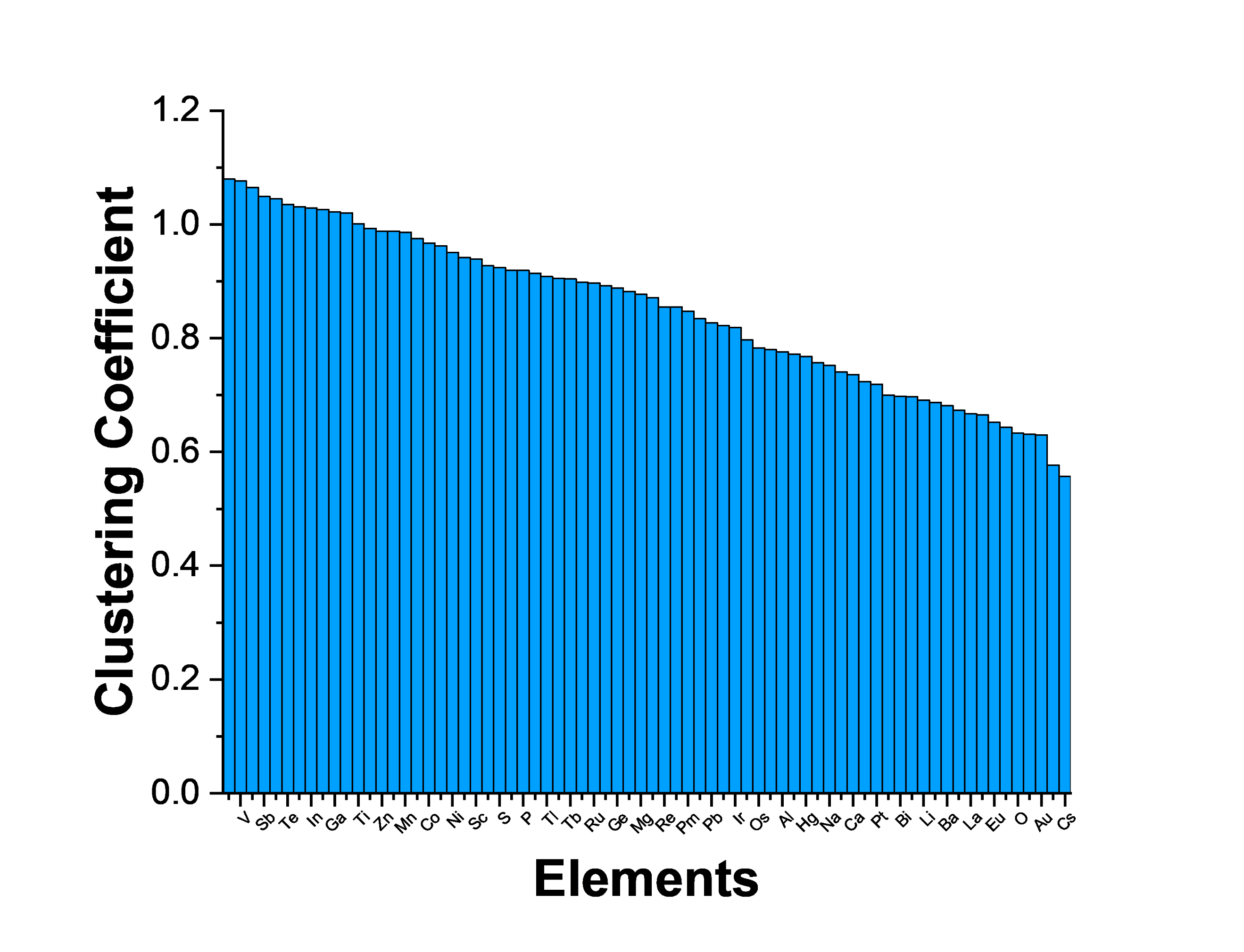


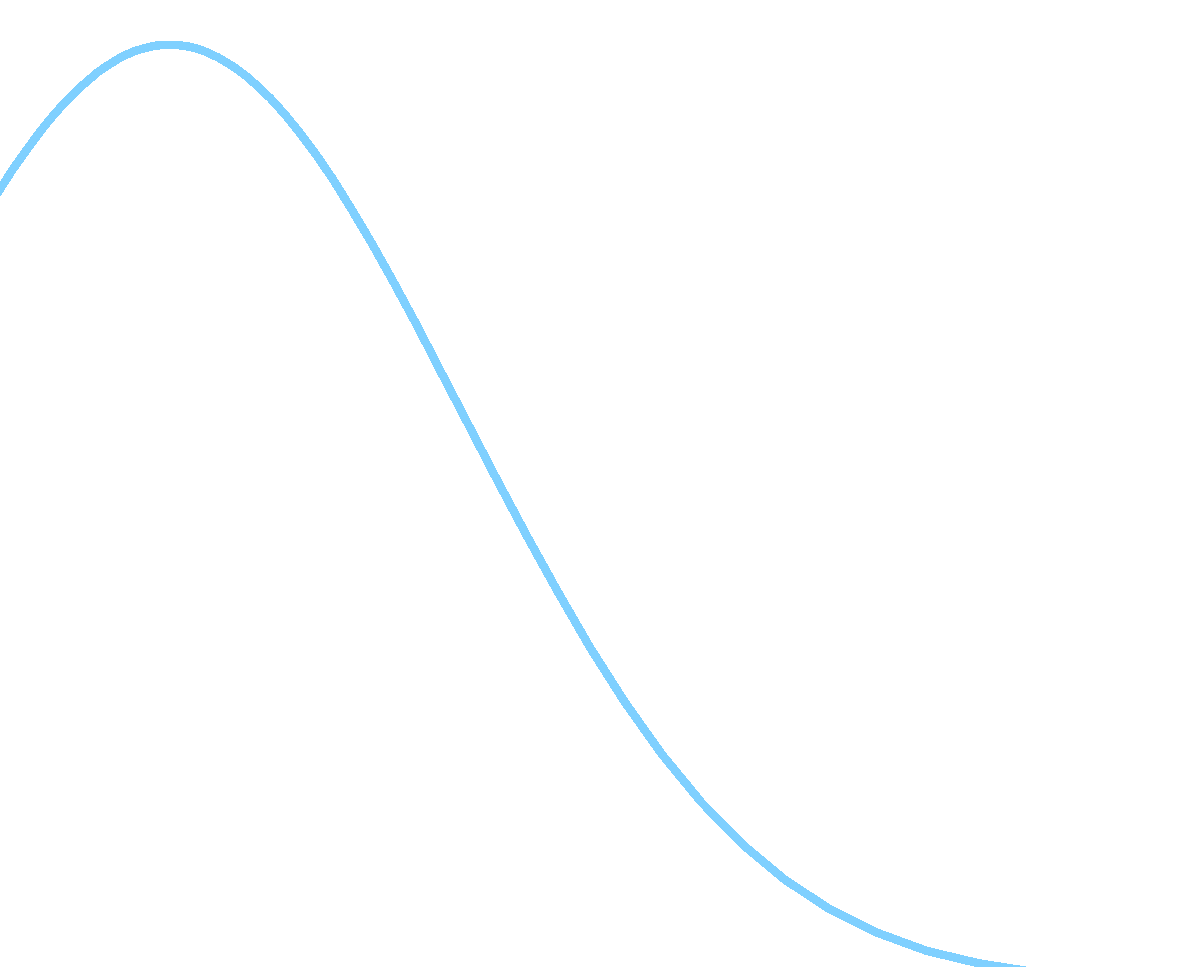

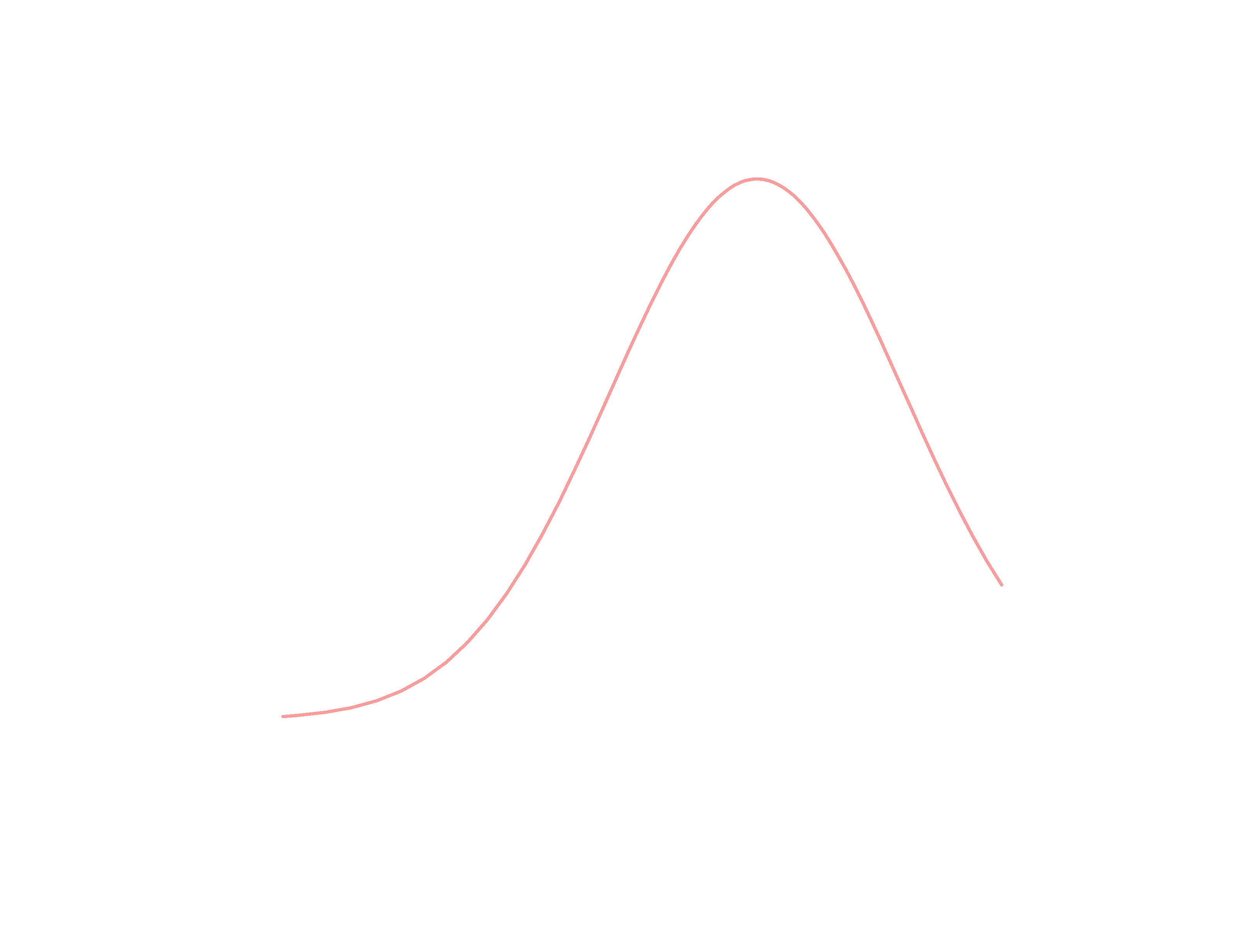

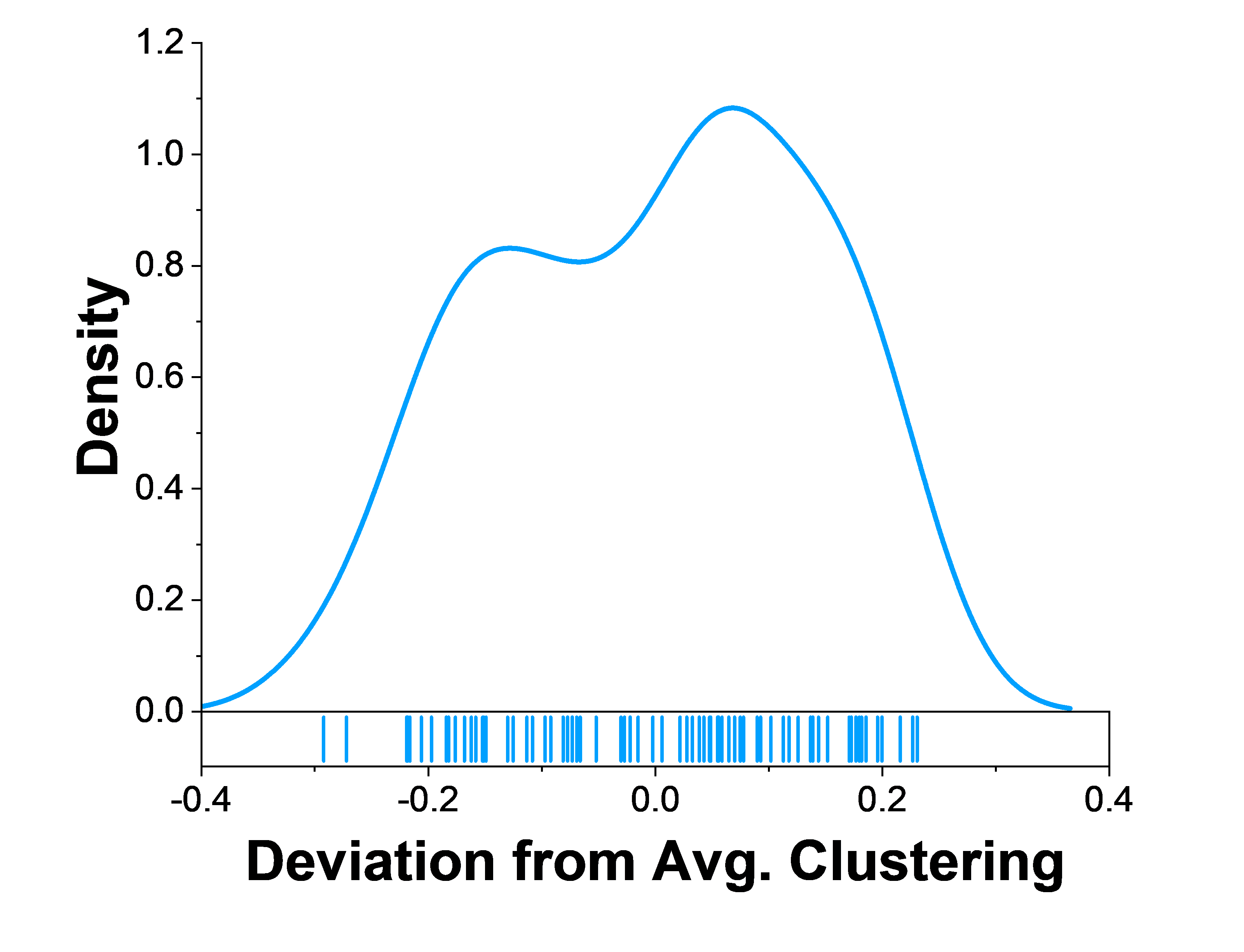


Hypoclustering

Hyperclustering

Average 0.85

-0.2

+0.2

Global Average

3.0

Global Average

0.85

**b**

**c**

**e**

**f**

**d**


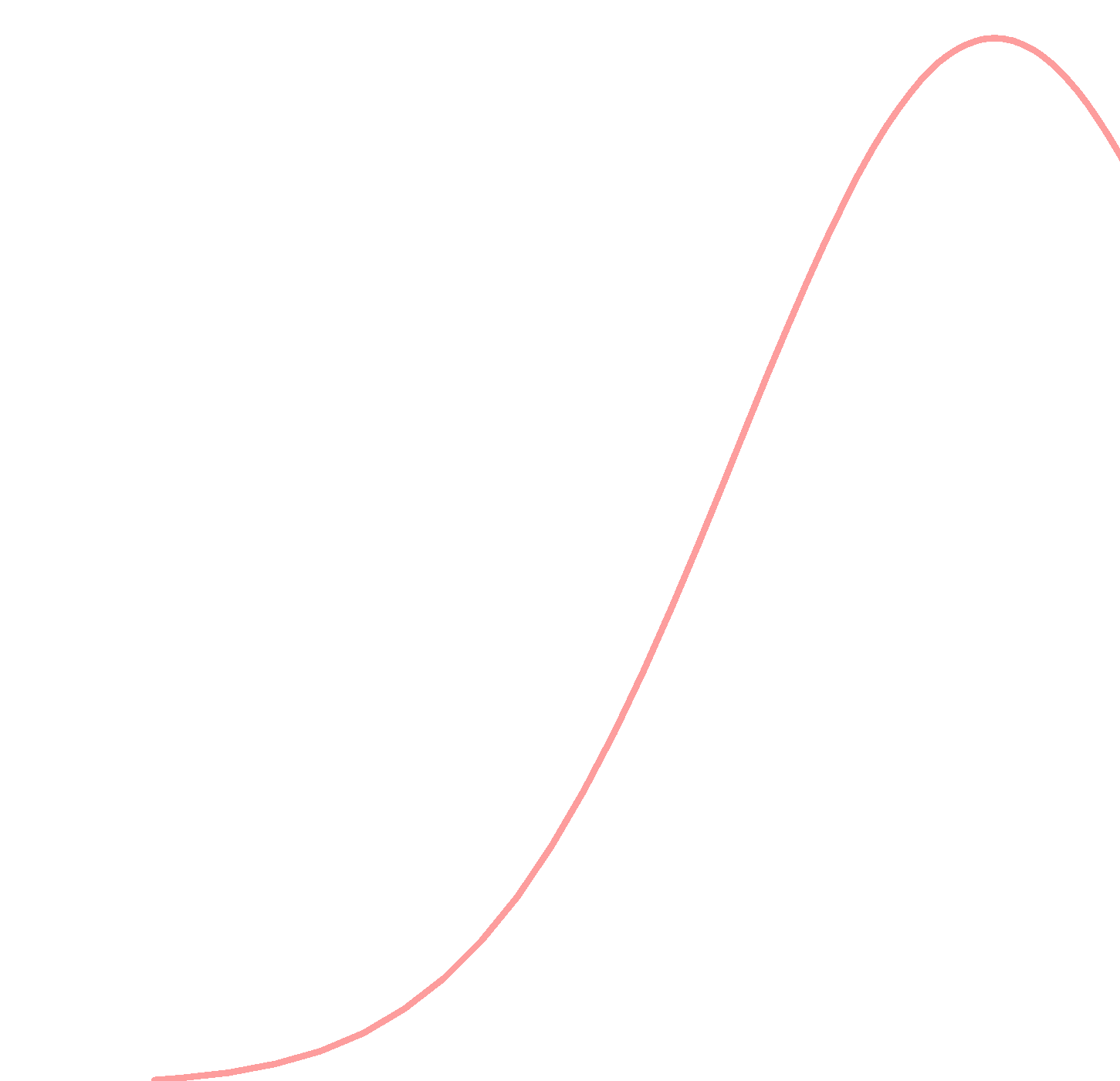

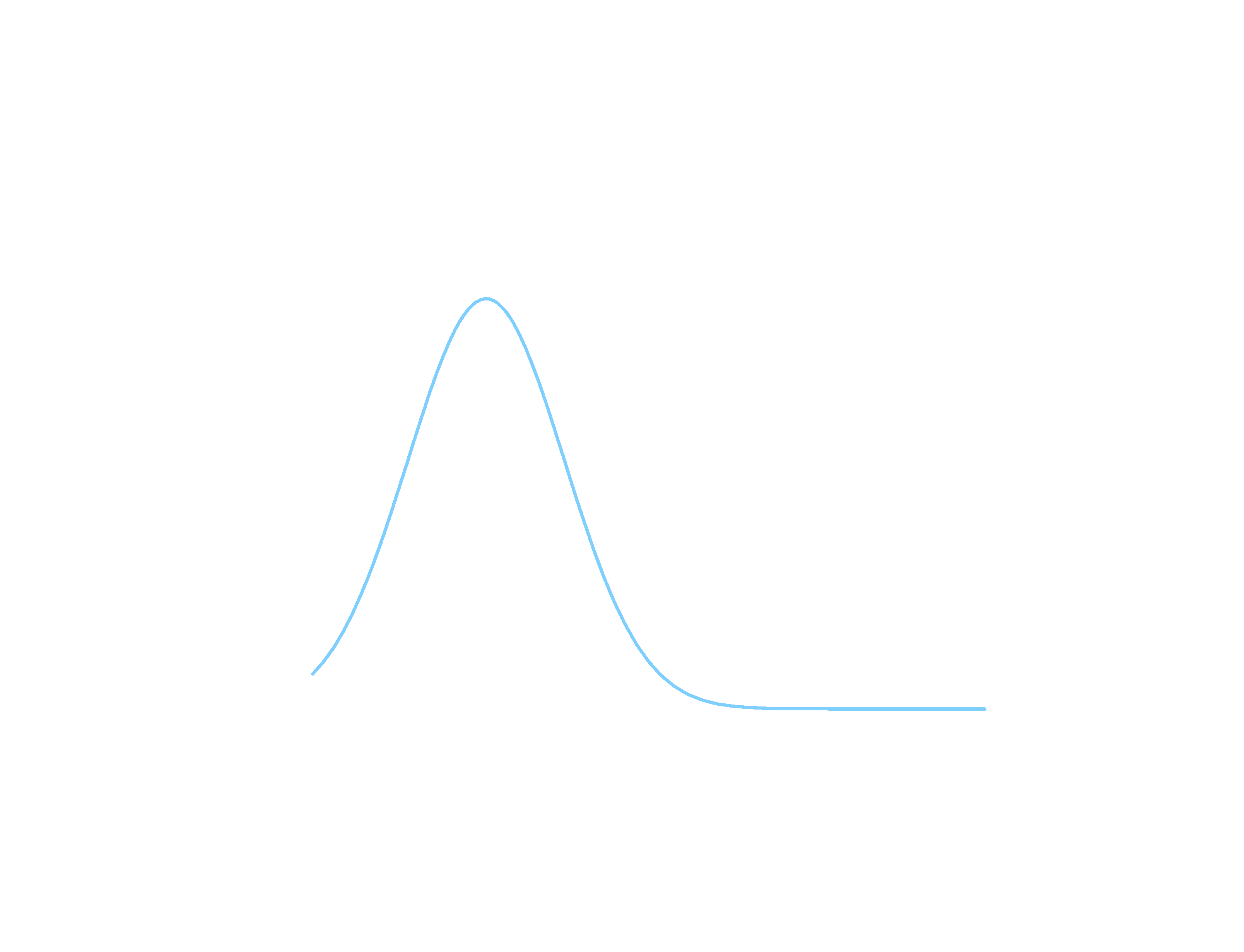

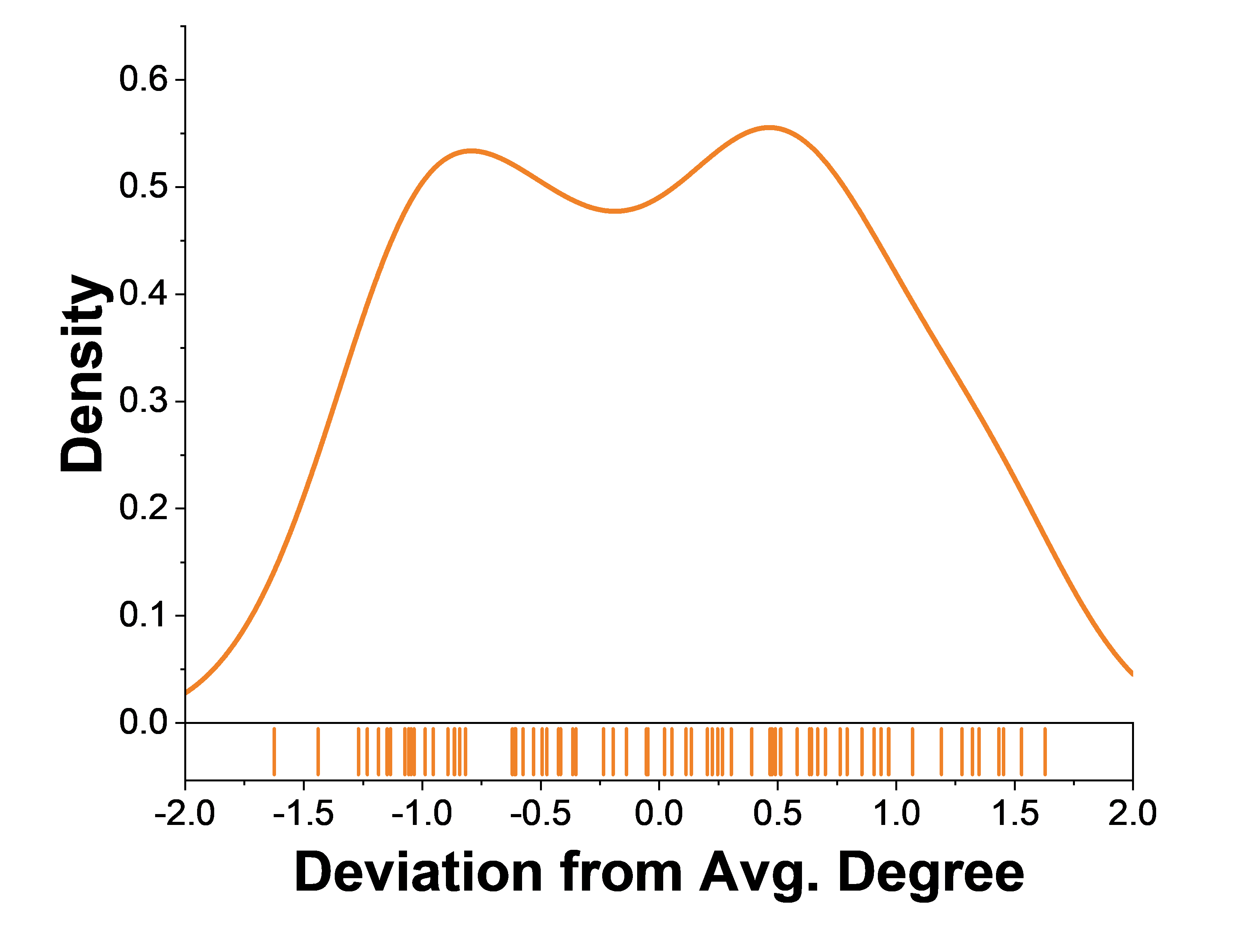


Hypoconnectivity

Hyperconnectivity

Average 3.0

-0.5

+0.5


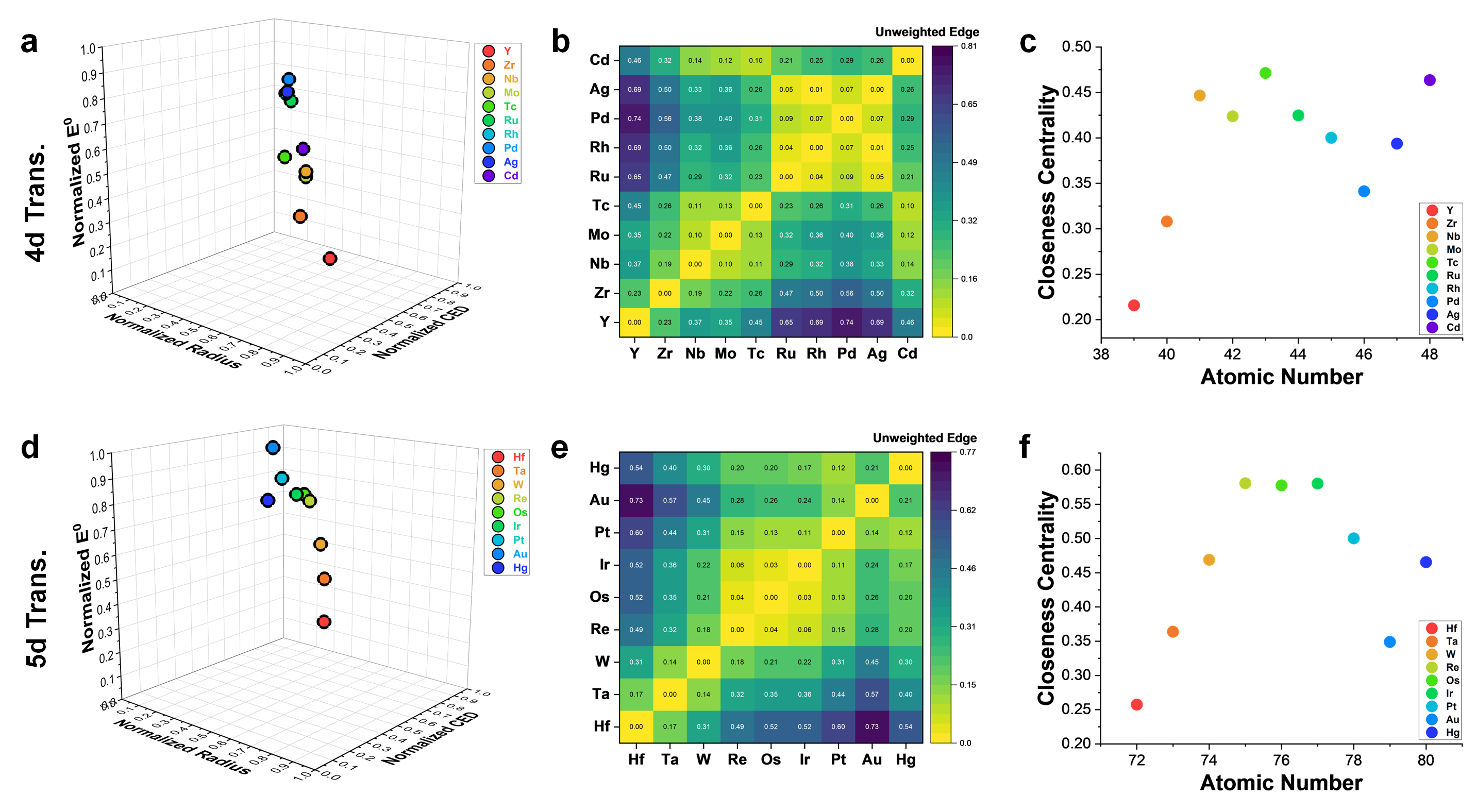


**Figure S5.** (a) Preferential Interactivity Parameter of 4d transition metals. (b) Calculated closeness centrality values of each 4d metals binary combination. (c) Unweighted edge heatmap matrix of 4d transition metals. (d) Preferential Interactivity Parameter of 5d transition metals. (e) Calculated closeness centrality values of each 5d metals binary combination. (f) Unweighted edge heatmap matrix of 5d transition metals.
